# Supplementary material for: A Thematic Synthesis Considering the Factors which Influence Multiple Sclerosis Related Fatigue during Physical Activity
Source: Behav Sci (Basel). 2019 Jul 1;9(7):70. doi: 10.3390/bs9070070 (PMC6680722; doi:10.3390/bs9070070)
Supplement: Supplementary file 1 [file behavsci-09-00070-s001.pdf]

## The Analytical Demographic and Methodology Characteristics of Included Studies

| STUDY | QUALITATIVE METHOD | PARTICIPANTS | SAMPLING & SETTING | DATA COLLECTIONS | STUDY AIMS | KEY THEMES |
|---|---|---|---|---|---|---|
| **1. Barnard, 2018** | Semi-structured interviews | 18 (13 F/5 M) PwMS<br>Sub type not given<br>Mean age: 50.3 years<br>Age range:32-68 years<br>Age range with illness: 4-42 years/ mean time: 9.39 years<br>Median: MS diagnosed 6 years ago<br>Participants level of disability (PDDS score): Mild 11/18, Moderate 3/18 and Severe 4/18 | Sampling: if they had attended a particular intervention<br>Time between intervention and interview:3-4.5 years<br>Setting: the interviews conducted via phone or Skype from a private office<br>Study geographical location: Victoria, Australia | Semi-structured interviews<br>Duration: not given<br>The questions identified the effect of healthy lifestyle for people with MS | To better understand barriers and enables to adherence a healthy behaviour in people with MS | Lifestyle factors<br>1) Diet,<br>2) Exercise<br>3) Stress reduction<br>4) Smoking<br>& Adherence Strategies/ Barriers /Advantages |
| **2. Borkoles 2007** | Semi-structured interviews | 7 (4 F/3 M) patients with MS<br>Sub type not given<br>Mean age: 47.1 years<br>Age range: 34-65 years<br>Mean time with illness: 16.3<br>EDSS: 4-6 | Sampling: purposing eligibility: MS categorised between 4-6 on EDSS<br>Setting: not given<br>Study geographical location: West Yorkshire, UK | Semi-structured interviews lasted 25-45min<br>General lifestyle questions & to describe their experiences of exercise since diagnosed MS<br>Then followed participants checking transcript and 1/7 further interviewed by phone | Examine the lived experiences related to exercise in people diagnosed with MS | 1) MS & disability<br>2) Participation in exercise<br>3) Meaning of exercise<br>4) Exercise, environment and social support |
| **3. Brown, 2012** | Focus group & in-depth interview | 8 (5 f/3 M)<br>Mean age not given<br>Age range: 47-66 years<br>Age range with illness 1-30 years/ mean time: 16,87 years<br>Sub type: 4 PP, 2 PR, 2 RR | Sampling:>18 years, self-reported diagnosis, availability to contribute verbally in focus group, English language, prior awareness of aquatic exs<br>Setting: MS Society of Canada, Manitoba Division | 1 semi-structured individual interview lasted 40 min and a focus group lasted 65 min<br>The focus group questions identified the follows:<br>1) What barriers face PwMS to participating in AF programs in Winnipeg? | To identify factors that facilitate or impede participation in Aquafitness programme (AF) by individuals with MS living in Winnipeg | 1) Benefits of AF Encourage Continued Participation / 2&3) Accessibility of the Environment to the Needs of PwMS./ 4) Pool Staff Attitudes and Knowledge Facilitating AF Participation./ 5) Lack of Support May Restrict Participation / 6) / 7) |

| | | | | | | |
|---|---|---|---|---|---|---|
| | | | Study geographical location: Winnipeg, Manitoba, Canada | 2) What facilitators help PwMS to participate in AF programs | | Participating in AF Requires Negotiating Fears |
| **4. Clarke, 2015** | Focus groups | 14 (no gender data) patients with MS<br>Sub Type: 1 RR, 3 SP, nothing given for the 10 participants<br>Mean age: 53.9<br>Mean time since diagnosis: 10.3<br>Three different groups: immediately, 5 & 4 months after the intervention | Sampling: individuals with MS who use at most one stick to walk outdoors<br>Setting: in hotels and MS Society building<br>Study geographical location: Limerick, Scotland | 1 focus group with open questions lasted 45min<br>Questions about decision to participate in community group exercise programme, previous experiences and outcomes from exercise<br>Transcripts were sent to the participants: 9 of them confirmed the moderator's interpretation | To explore the perceptions of participants (individuals with MS) of a 10 weeks community group exercise programme | 1) Positive and negative effects of the intervention<br>2) What aspect of the programme did they enjoy<br>3) If they will continue to exercise when the programme is completed |
| **5. Crank, 2017** | Focus groups & semi-structured telephone interviews | 33 participants totally with mean age 47.6 years<br>29 participants took part in focus groups (23 F/6 M)<br>Mean age 48.8 years<br>Mean time since diagnosis 8.8 years / EDSS 3.8<br>4 interviewed by telephone (3F/1M)<br>Mean age 48.8 years<br>Mean time since diagnosis<br>9.7 years / EDSS 3.0<br>Sub type: not given | Sampling:33/54 individuals of the participants in a 12 weeks exercise programme (within 6 months of completing the programme), with mild to moderate MS<br>Setting: University exercise science department close to the recruiting hospital.<br>Study geographical location: Sheffield | 6 focus groups (2-8 participants) lasted 60-80min<br>& telephone interviews lasted ~30min<br>Open-ended questions were used to guide the focus group and interview discussions | Exercise perceptions and experiences in PwMS before, during, and after participation in 12-weeks personal tailored exercise program. | 1) Transition to inactivity<br>2) Lack of knowledge and confidence<br>3) Positive exercise experience<br>4) Perspectives on exercise adherence |
| **6. Dodd, 2006** | In-depth semi-structured interviews | 9 (7 F/2 M) MS patients<br>Mean age: 45.6 years<br>Age range: 27-61 years<br>Mean time since diagnosis: 6 years<br>Sub type: not given | Sampling: individuals with MS with mild to moderate disability who participated in a 10-week progressive resistance exercise programme (PRE) | Semi-structured interviews lasted about 30 min for each participant<br>Evaluation of positive and negative perceptions of participating in PRE programme and to identify | To explore the outcomes (positive & negative) of a progressive resistance exercise programme in adults with MS, | Positive outcomes of progressive resistance exercise: Physical, Physiological, Social<br>Factors important for programme competition: Extrinsic & Intrinsic |

| | | | | | | |
|---|---|---|---|---|---|---|
| | | | Setting: place at their home (5) or in a quiet room at the gymnasium /after the final exercise session<br>Study geographical location: Australia | facilitators and barriers in the completion of the programme. | facilitators or barriers in the completion of the programme. | |
| **7. Horton, 2015** | Semi-structured interviews | 5 MS patients (1 F & 4 M)<br>Age range 45-70 years<br>Mean age 57.4 years<br>Average age diagnosed with MS 44 years old<br>5 MS spouses (4 F & 1 M)<br>Age range 44-69 years<br>Mean age 56.8 years<br>All participants were Caucasian<br>Sub type: not given | Sampling: individuals with MS with disability status between 4-6 on the EDSS and their spouses<br>Setting: in a private room with one interviewer<br>Study geographical location: not given | 1 Semi-structured interview lasted approximately 60 min for each participant separately (from 40 to 75min) / open-ended questions, careful listening and of the interviewer<br>Included questions about their current exercise regime, overcoming barriers to exercise, and impact on their quality of life. | To investigate the exercise experiences of individuals with MS and the extent to which these experiences affect, or are affected by, their spousal relationship. | 1) Maintaining independence<br>-Effect of MS on the spousal relationship/ physical functioning<br>2) Overcoming isolation<br>3) Negotiating if exercise is worth it (Managing energy levels / A growing acceptance of exercise) |
| **8. Kayes, 2011** | Semi-structured interviews | 10 MS patients (7 F/3 M)<br>Mean age: 44.3 years<br>Age range: 34-53 years<br>Age range since diagnosis: 3-17 years<br>Mean time since diagnosis: 8.8 years<br>Sub type: CP 3, SP: 3, RR: 4<br>On the GNDS ranged between 1 to 31 | Sampling: individuals with definite diagnosis of MS and able to communicate with the researchers. Selected with diversity in age, gender and type of MS<br>Setting: in their home<br>Study geographical location: New Zealand | Semi-structured interviews lasted from 60 to 90 min<br>Open-ended interview where they explored patients' current engagement in physical activity, barriers and facilitators to physical activity & perceived impact of physical activity | To conduct a qualitative inquiry exploring PwMS engagement in PA, to develop an in-depth understanding of the barriers and facilitators to engagement | Management strategies<br>Concerns about others<br>Social support<br>Advice from and access to health professionals<br>Lazy or MS? / Self-efficacy<br>Impairment /External factors<br>Personal resources |
| **9. Learmonth, 2012** | 2 Focus groups | 14 MS patients (10 F/4 M)<br>Mean age: 59.6 years<br>Age range: 40-68 years<br>Age range since diagnosis: 4-33 years | Sampling: individuals with diagnosis of MS, an EDSS score of 5-6.5 and cognitive score of 24 or over on the Mini Mental State Examination<br>Setting: not given | 2 focus groups, lasted approximately 60min, held in the last week of the exercise programme. At Site B the moderator added some questions to the interview schedule in order | Investigated: 1) participants' experience and perspective of a group exercise intervention (GEI); 2) positive and | Three key themes were identified from the two focus Groups:<br>1) The exercise class<br>2) Benefits of the class<br>3) Barriers to exercise. |

| | | | | | | |
|---|---|---|---|---|---|---|
| | | Mean time since diagnosis: 16.9<br>EDSS range score 5-6.5<br>Sub type: not given | Study geographical location: Ayrshire & Arran, Scotland | to further explore barriers to exercise and exercising in a group. | negative outcomes from participating in GEI; 3) factors which facilitated or inhibited people moderately affected with MS to participate in GEI | |
| **10. Plow, 2009** | Qualitative pilot study- Semi-structured interviews | 13 MS patients (11 F/2 M)<br>Mean age: 46.7 years<br>Age range: 18-68 years<br>Age range since diagnosis: 1-43 years<br>Mean time since diagnosis: 12.2 years<br>Sub type: SP: 2, RR: 9, Unknown: 2 | Sampling: purposing eligibility: MS confirmed diagnosis of MS and the ability to walk with or without an assisting device, selected with a heterogeneity in age, education, and disability and they were willed to tell their story<br>Setting: not given<br>Study geographical location: US | Semi-structured interviews, lasted approximately 1.5 hours, took place between the post-test and follow up assessments of the clinical trial.<br>Participants described how coped with their diagnosis of MS initially and at present, their general beliefs and experiences from exercise programme.<br>After completion of interviews participants classified by activity level as: active, sometimes active, or inactive. | To understand how the person-environment interaction and coping with MS influence PA behaviour from the perspective of people with MS. The secondary aim of the study was to explore the relevance and utility of SCT and TMSC in understanding PA behaviour among persons with MS. | Investigated similarities and differences between active, sometimes active, and inactive participants' behaviour associated with their barriers to PA (included symptoms, accessibility, social obligations), and their facilitators of PA (included self-regulation, barrier self-efficacy & positive coping styles) |
| **11. Schneider, 2018** | Semi-structured personal interviews | 7 women with MS<br>Mean age: 50.4 years<br>Age range: 41-55 years<br>Age range since diagnosis: 2-9 years<br>Mean time since diagnosis: 5 years<br>Sub type: PP: 3, RR: 4<br>All the participants were Caucasian | Sampling: purposing eligibility: females between 40-55 years of age who had been formally diagnosed with either PP or RR multiple sclerosis, a minimum of two years prior.<br>Setting: 6 of the interviews in their home & 1 by phone<br>Study geographical location: Not given | Questionnaire was completed prior to their interview.<br>Semi-structured interviews lasted from 19 to 53 min (mean time 41 min) were consisted of 27 open-ended questions about current level of PA, the limitations they face when accessing PA, associated stressors, coping mechanisms, and | To examine the specific coping strategies, they used when managing their MS, in relation to their participation in physical activity. | Self-management, treatments, and personal attitude. |

| | | | | | | |
|---|---|---|---|---|---|---|
| | | | | their perceived quality of life & field notes. | | |
| **12. Smith, 2009** | 3 Semi-structured interviews | 10 (8 F/2M) with MS<br>Mean age: 46.4 years<br>Age range: 32-61 years<br>Age range since diagnosis: 1-40 years<br>Mean time since diagnosis: 13.1 years<br>Sub type: RR | Sampling: individuals with confirmed diagnosis of MS, who had experienced changes in energy levels but could still walk short distances independently.<br>Setting: Not given<br>Study geographical location: New Zealand | 3 semi-structured interviews contacted before, at mid-way and after intervention. Participants were encouraged to tell what it is like for them to have MS (avoid using the term "fatigue and exercise").<br>Duration of interviews: not given | To explore in depth the influence of an eight-week exercise programme on fatigue perceptions in people with MS. | 1) Perceived control (Possession, Power struggle, Power sharing)<br>2) Listening to your body<br>3) Reaching the edge<br>4) Nature of tiredness<br>5) Exercise outcomes |
| **13. Smith, 2011** | Interviews | 9 Women with MS<br>Mean age: not given<br>Age range: 28-70 years<br>Age range since diagnosis: less than 1 year to 30 years<br>Mean time since diagnosis: not given<br>Sub type: SP: 3, RR: 5, 1 unknown<br>Most of the participants were New Zealand European/Pakeha | Sampling: individuals with diagnosis of MS, who experienced a decline in energy levels but able to walk short distances independently & exercised within their community at least once weekly over the last two months.<br>Setting: at their home or another convenient location<br>Study geographical location: New Zealand | Interviews lasted 50 min to 2h (with breaks).<br>Interviews generally began with; "I'd like you to tell a story that helps me understand what it is like for you to have MS" and participants encouraged to give further information about exercise and fatigue. | How does fatigue influence community-based exercise participation in PwMS (how PwMS described fatigue, and how experiences embedded in these descriptions influenced participation in community-based exercise programme). | The nature of the beast<br>Choosing to exercise:<br>1) "Wellness philosophy" supported exercise<br>2) A "related goal" significant to the management<br>3) Perception that "control is possible" |
| **14. Smith, 2013** | Focus group, interviewed via telephone, interviewed face to face | 6 physiotherapists (PTs), 3 occupational therapists (OTs), 3 MS society support workers and 3 consultant neurologists<br>All the participants were from New Zealand | Sampling: HCPs experienced practitioners with MS (if they had a regular contact with and provision of active advice/intervention for people with MS over a 2-year period) | (PTs) & (OTs) participated in focus group discussions, MS society support workers interviewed via telephone and consultant neurologists interviewed face to face.<br>Duration: not given<br>Open-ended questions schedule included: to tell | To explore, describe, and interpret the experiences of HCPs who provide interventions and advice for people with MS-related fatigue in order to identify possible | Nature of fatigue<br>Professional challenges:<br>1) Barriers to implementation<br>2) Stirring conflict<br>3) Modifying roles<br>Demanding creativity:<br>1) Challenging science<br>2) Mind body |

| | | | | | | |
|---|---|---|---|---|---|---|
| | | | Setting: not given<br>Study geographical location: New Zealand | about their involvement with PwMS, suitability of exercise with fatigue in people with MS, how they advise/counsel or treat PwMS, and their role in fatigue management in PwMS. | strategies that might assist with this process. | |
| **15. Smith, 2015** | Interviews | 18 men with MS<br>Mean age: not given<br>Age range: 36-68 years<br>Age range since diagnosis: 3 to 21 years<br>Mean time since diagnosis: not given<br>Sub type: SP: 3, RR: 10, & PP: 5.<br>Most of the participants were New Zealand European ethnicity | Sampling: Male PwMS who experienced energy changes but walked independently & exercised in community at least once per week within the last 2 months<br>Setting: 2 by telephone, 1 at the local MS society & 15 at their home (2 participants had a follow up interview)<br>Study geographical location: New Zealand | Interviews lasted no longer than 1 h.<br>Participants asked to: tell what it's like to have MS, their experience of fatigue; how do they manage fatigue and exercise, how choose that particular exercise, how they feel when do exercise or don't exercise, and what it means to be able to exercise." | How does fatigue influence community exercise participation in men? Can we identify any potential differences between men's and women's responses to fatigue that might help guide future lines of inquiry? | Complex expressions of fatigue<br>Engaging in exercise and goal adjustment<br>Emotional responses to fatigue and exercise |
| **16. Stennett 2018** | Semi-structured interviews | 16 individuals with MS (12 F/4M)<br>Mean age: 61.3 years<br>Age range: 47-72 years<br>Age range since diagnosis: not given<br>Mean time since diagnosis: not given<br>Sub type: SP: 4, RR: 2, PP: 5 & Unknown:5 | Sampling: 2 Mildly, 6 Moderately & 8 Severely affected PwMS living in the community who completed a prior Delphi study<br>Settings: in a location of the participants' choice (home, a room at the university, or at MS therapy centres)<br>Study geographical location: London | Interviews lasted 45 to 60 min<br>Included questions like: What does the term exercise and physical activity mean to you? What are your thoughts on the physical activities selected by people with MS? What are your thoughts on the reasons why these physical activities were selected? | To explore the everyday meanings of exercise and physical activity in community dwelling people with MS. | "A Type of Movement", "The Impact of Exercise and Physical Activity", "It Changes", "Sense of Loss" and "Coping with MS". |
| **17. Stuifbergen, 1997** | Semi-structured interviews | 13 individuals (10 F/3 M)<br>Mean age: 44.6 years<br>Age range: 35-63 years | Sampling individuals, who had been diagnosed with MS for more than 1 | Conducted 2 interviews lasted 60-90 min | a) conceptualization of fatigue in PwMS, | The experience of fatigue<br>Antecedent factors of fatigue |

| | | | | | | |
|---|---|---|---|---|---|---|
| | | Age range since diagnosis: 2 to 47 years<br>Mean time since diagnosis: 13.9 years<br>Sub type: not given<br>12 were White and 1 Black | year, were between 20 to 70 years old and not experiencing other major health problems or pregnancy<br>Settings: not given<br>Study geographical location: not given | On the 1st the participant told his own story, how experienced fatigue in his life.<br>On the 2nd clarified transcribed data from the 1st interview | b) antecedents of fatigue in PwMS,<br>c) behaviours which enable PwMS manage fatigue, and<br>d) factors influence PwMS to selecte and use of strategies for managing fatigue | Self-care strategies used to manage fatigue<br>Influences on selection and initiation of self-care strategies |
| **18. Turpin, 2015** | Interviews with semi-structured and structured component | 31 patients with MS (23 F/ 8 M)<br>Mean age: 51.3 years<br>Age range: 25-70 years<br>Age range since diagnosis: 1 to 25 years<br>Mean time since diagnosis: 10.3 years<br>Mean years since symptom commencement:16.8 (range:3-46)<br>Sub type: RR: 21, SP: 4, PP: 4, Unknown: 2 | Sampling: participants 18 from USA and 13 from Australia with direct experience of MS fatigue<br>Settings: not given<br>Study geographical location: Chicago, IL & Brisbane, Queensland in Australia | Conducted interviews lasted 60-150 min (mean length 85min)<br>The interviews had 3 parts:<br>1st Asked: "How has MS fatigue changed your everyday routine and activities?"<br>2nd Participants asked about 15 common fatigue management strategies to sort them according to whether they used each strategy regularly, sometimes, or never<br>3rd Asked to provide specific demographic information and complete two standard MS measures | How people managed MS fatigue in the context of their unique daily lives, exploring what they did and why, rather than taking a particular theoretical perspective towards the issue. | Strategies categorized as "used regularly": Plan and organize the day/ Avoid heat and humidity /Break up activities over the course of the day or over several days & Sit to do an activity.<br>Strategies categorized as 'used sometimes: Ask someone for help, Try to make activities simpler, Delegate an activity to someone else.<br>Strategies categorized as 'never used': Use mobility devices, Use gadgets to make jobs easier, Do exercise to build endurance. |
| **19. Twomey 2010** | Semi-structured in-depth interviews | 8 patients with MS (6 F/ 2 M)<br>Mean age: 42.8 years<br>Age range: 29-55 years<br>Age range since diagnosis: 1 to 20 years<br>Mean time since diagnosis: 10.1 years<br>Sub type: RR: 3, SP: 2, Unknown: 2, Benign:1 | Sampling: 8/11 individuals with MS with experience of fatigue related to MS and participated in a fatigue management exercise programme<br>Settings: not given<br>Study geographical location: Ireland | Interviewed 1 month after the completion of fatigue management programme.<br>Interviews lasted 45 to 90 min<br>Participants were asked about their experiences of being part of a group, their perceptions of the programme content, what | To describe the subjective experiences of participating in a fatigue management programme for people with MS. | 1) Participant's experiences of ownership, active participation and empowerment.<br>2) Legitimisation of fatigue and validation.<br>3) Experiences of support, having a shared voice and shared experiences. |

| | | | | | | |
|---|---|---|---|---|---|---|
| | | | | changes they made as result of participating in the programme, and their suggestions for future programmes | | The outcomes of the programme: lifestyle and occupational changes, altered thinking about fatigue and the development of social supports. |

**<u>Abreviations:</u>**
**PA**: physical activity
**EDSS**: *The Kurtzke Expanded Disability Status Scale; Kurtzke, 1983*
**GNDS**: *Guys Neurological Disability Scale*
**PP**: *Primary Progressive*
**PR**: *Progressive Relapsing*
**RR**: *Relapsing Remitting*
**CP**: *Chronic Progressive*
**SP**: *Secondary Progressive*
**SCT**: *Social Cognitive Theory*
**TMSC**: *Transactional Model of Stress and Coping*

## Appendix II The Completed Modified COREQ Soundy et al [3] (Studies 1/19 -10/19)

| **Domain 1: Research team and reflexivity** | **Scoring** | **Barnard, 2018** | **Borkoles 2007** | **Brown 2012** | **Clarke 2015** | **Crank 2017** | **Dodd 2006** | **Horton 2015** | **Kayes 2011** | **Learmonth 2012** | **Plow 2009** |
|---|---|---|---|---|---|---|---|---|---|---|---|
| | | **Study 1** | **Study 2** | **Study 3** | **Study 4** | **Study 5** | **Study 6** | **Study 7** | **Study 8** | **Study 9** | **Study 10** |
| **Personal characteristics** | | | | | | | | | | | |
| 1. Interviewer/facilitator Which author/s conducted the interview or focus group? | If they have identified give a point, if they haven't give 0, if you unclear type U. | 1 | 0 | 0 | 1 | 0 | 0 | 0 | 1 | 1 | 1 |
| 2. Experience and training What experience or training did the researcher have? | Where experience is clearly detailed or a detailed reference to training OR experience is made a point is given. IF unclear or absent give zero | 1 Experience but not training | 0 | 0 | 0 | 1 Trained in qualitative research techniques | 1 | 1 Experience, did not mention formal training | 0 | 0 | 0 Not given |
| **Relationship with participants** | | | | | | | | | | | |
| 3. Relationship established Was a relationship established prior to study commencement? | Give a point if details of how the researcher or person who undertook qualitative data collection met individuals, identified any previous relationship. Where this is unclear type U. Where this information is absent type 0. | 0 No prior relationship | 0 No relationship | 0 No relationship | U | U Did not directly involved in the exercise trial but worked in exercise research | 0 No relationship | 1 | U They were selected from the population of a larger study | 0 The interviewers were unknown to the participants | U Not given |
| 4. Participant knowledge of the interviewer. What did the participants know about the researcher? e.g. personal goals, reasons for doing the research | A point is scored where information about what the participants knew about the research, they were being invited to participate in was mentioned. This includes sending background information and study information sheets. Score zero where this information is absent and U where this information is unclear | 1 Invitations were sent to the participants who took part in a MS health behaviour | 1 Information provided on a letter | 1 Information provided with brochures, leaflets, and by local | 1 Information provided on leaflets and on the introduction of the | U | 0 | 1 3 of the participants (& 3 their spouses) involved on the lead author's exercise program | U It is a part of a larger qualitative study and the sample was been informed for the possibility | U Participants were part of a larger randomised controlled study and recruited through the Managed Clinical Network for MS | U |

| | | | | | | | | | | | |
|---|---|---|---|---|---|---|---|---|---|---|---|
| | | intervention | | MS chapter | focus group | | | provided the initial contact | to participate in this study | within NHS Ayrshire and Arran | |
| 5. Interviewer characteristics What characteristics were reported about the interviewer/facilitator? e.g. Bias, assumptions, reasons and interests in the research topic | Score a point where information about the characteristics of the interviewer, their bias, interests or reasons for participating in research are identified. Score zero where this information is absent. Score u where this information is unclear. | 1 One of the authors but had no involvement in recruitment, interviewing or data analysis | 0 | 0 | U | 0 | U | 1 The lead author's former involvement in an exercise program | 0 | 1 The authors involved in a larger randomised controlled study which investigated the effects of exercise in MS patients | U |
| **Total Score for Domain 1.** | | **4/5** | **1/5** | **1/5** | **2/5** | **1/5** | **1/5** | **4/5** | **1/5** | **2/5** | **1/5** |
| | | | | | | | | | | | |
| **Domain 2: study design** | | | | | | | | | | | |
| **Theoretical framework** | | | | | | | | | | | |
| 6. Methodological orientation and theory. What methodological orientation was stated to underpin the study? e.g. grounded theory, discourse analysis, ethnography, phenomenology | Score a point where the paradigm and methodology are given. Score zero where both aspects are missing. Score U where this information is unclear. | 1 A pragmatic evaluation | 1 Interpretative Phenomenological Analysis | 1 Phenomenology | 1 A pragmatic evaluation approach | 1 Constructivist | 0 | 0 | 1 Grounded theory | 0 | 1 Qualitative design, part of a mixed methodology study. Modified Analytical Induction (interview analysis) |
| 7. Non-participation How many people refused to participate or dropped out? Reasons? | Score a point where the number and the reason or attempts to identify the reason are given (e.g., a point is score if they say participants would not give a reason for non-participation). Score zero where this information | 0 Not given | 1 Not refused. 9 did not fit the eligible criteria | 1 8/14, 1 lack of child care, 1 lack of transpo | U Lack of information | 1 33/54 who participated in the trial | 1 | 0 | 1 Researchers selected them from a broader sample | U The authors excluded potential participants if they experienced an exacerbation | 1 Did not complete the quantitative component without |

| | | | | | | | | | | | |
|---|---|---|---|---|---|---|---|---|---|---|---|
| | is absent and score u where this information is unclear. | | | rtation, 4 not given reason | | | | | | of their MS symptoms within the period of the trial | given the reasons |
| **Data collection** | | | | | | | | | | | |
| 8. Interview guide Were questions, prompts, guides provided by the authors? Was it pilot tested? | Score a point where testing of the interview script is identified either as a pilot or as a way to determine the content and accuracy of items used. Score a point where consideration to the derivation of questions have come from. | 0 Not given | 1 Interview schedule provided | U | 1 Additional probes used to supplement the focus groups questions | 1 | 1 | 1 | 1 | 1 | 1 |
| 9. Field notes - Were field notes or reflective diary made during and/or after the interview or focus group? | Score a point where field notes are identified. Score zero where field notes are not identified. Score U where this information is unclear. | 0 Not given | 1 | U | 1 | 0 | 0 | 0 | 0 | 1 | 0 |
| 10. Data saturation Was data saturation discussed? | Score a point where saturation of data is considered. Score a point where another form of sample size reference is made. Score U where this information is unclear. | 1 Yes | 0 | U | 0 | 1 | 1 | 0 | 1 | 0 | 1 |
| **Total for Domain 2** | | **2/5** | **4/5** | **2/5** | **3/5** | **4/5** | **3/5** | **0/5** | **4/5** | **2/5** | **4/5** |
| | | | | | | | | | | | |
| **Domain 3: analysis & findings** | | | | | | | | | | | |
| **Data analysis** | | | | | | | | | | | |
| 11. Description of the coding tree. Did authors provide a description of the coding tree? | Score a point if an audit trail is given. Score a point if a coding tree is mentioned or score a point if another technique is mentioned that provides a way to structure the information gained. | 1 | 0 | 1 | 1 | 1 | 1 | 1 | 1 | 1 | 1 Yes |

| | | | | | | | | | | | |
|---|---|---|---|---|---|---|---|---|---|---|---|
| 12. Derivation of themes Were themes identified in advance or derived from the data? | Score a point if data driven or theory driven coding is identified or if it is clear how analysis was determined | 1 Derived from the data | 1 Derived from the data | 1 Derived from the data | 1 Derived from the data | 1 Both | 1 Both | 1 Derived from the data | 1 Both | 1 Derived from data | 1 Both |
| **Reporting** | | | | | | | | | | | |
| 13. Clarity of minor themes Is there a description of diverse cases or discussion of minor themes? | Score a point if there is sub-detail for each major theme. Score no points where this information is absent or u if this information is unclear. | 1 | 0 Several themes were collapsed into one | 1 | 0 | 1 | 1 | 1 | 1 | 1 | 0 |
| **Total for domain 3** | | 3/3 | 1/3 | 3/3 | 2/3 | 3/3 | 3/3 | 3/3 | 3/3 | 3/3 | 2/3 |
| | | | | | | | | | | | |
| **Grand Total Score for studies 1/19 – 10/19** | | **9/13** | **6/13** | **6/13** | **7/13** | **8/13** | **7/13** | **7/13** | **8/13** | **7/13** | **7/13** |

# <u>Appendix II</u> The Completed Modified COREQ Soundy et al [3] (Studies 11/19 -19/19)

| Domain 1: Research team and reflexivity | Scoring | Schneider 2018 | Smith 2009 | Smith 2011 | Smith 2013 | Smith 2015 | Stennett 2018 | Stuifbergen 1997 | Turpin 2015 | Twomey 2010 |
|---|---|---|---|---|---|---|---|---|---|---|
| | | **Study 11** | **Study 12** | **Study 13** | **Study 14** | **Study 15** | **Study 16** | **Study 17** | **Study 18** | **Study 19** |
| **Personal characteristics** | | | | | | | | | | |
| 1.Interviewer/facilitator Which author/s conducted the interview or focus group? | If they have identified give a point, if they haven't give 0, if you unclear type U. | 1 | 1 | 0 | 0 | 0 | 1 | 0 | 1 | 1 |
| 2. Experience and training What experience or training did the researcher have? | Where experience is clearly detailed or a detailed reference to training OR experience is made a point is given. IF unclear or absent give zero | 0 Not given | 0 Not given | 0 | 0 | 0 | 1 Physiotherapist with special interest in Neurology | 0 | 1 A specialist in MS rehabilitation and research | 1 Occupational therapist |
| **Relationship with participants** | | | | | | | | | | |
| 3.Relationship established Was a relationship established prior to study commencement? | Give a point if details of how the researcher or person who undertook qualitative data collection met individuals, identified any previous relationship. Where this is unclear type U. Where this information is absent type 0. | 0 No relationship | 1 They were recruited from selected physio clinic and the local society via advertisement, but the study was part of a mixed method study | 0 No relationship | 0 | 0 | 0 Not given any relationship (the study followed a previous Delphi study) | 0 Not given | 0 No relationship | 1 Partially involved in the quantitative component of the study |
| 4. Participant knowledge of the interviewer. What did the participants know about the researcher? e.g. personal goals, reasons for doing the research | A point is scored where information about what the participants knew about the research, they were being invited to participate in was mentioned. This includes sending background information and study information sheets. Score zero where this information is absent and U where this information is unclear | 1 Informed by advertisements posted at a local neurologist's office | 0 Not given | 1 Informed by advertisement in a local MS society | 1 Detailed information sheets were either e-mailed or posted to the possible | 1 Received a detailed information sheet by email | U Participants from a prior Delphi study were asked for their willing to participate, but not given any other information | U Participants recruited through notices about the study in a local newslett | 1 The study was advertised through flyers and presentations, direct letters to participants of previous | 1 The aim of the study was presented verbally, and information |

| | | | | | | | | | | |
|---|---|---|---|---|---|---|---|---|---|---|
| | | | | | participants | | | er for MS patients | research who had agreed to be contacted for further | leaflets distributed |
| 5. Interviewer characteristics What characteristics were reported about the interviewer/facilitator? e.g. Bias, assumptions, reasons and interests in the research topic | Score a point where information about the characteristics of the interviewer, their bias, interests or reasons for participating in research are identified. Score zero where this information is absent. Score u where this information is unclear. | 1 | 0 Not given | 0 Not given | 0 Not given | 0 Not given | 1 The primary researcher: Interest in neurology and the others experienced with MS patients | 0 Not given | 0 Not given | 0 |
| **Total Score for Domain 1.** | | **3/5** | **2/5** | **1/5** | **1/5** | **1/5** | **3/5** | **0/5** | **3/5** | **4/5** |
| | | | | | | | | | | |
| **Domain 2: study design** | | | | | | | | | | |
| **Theoretical framework** | | | | | | | | | | |
| 6.Methodological orientation and theory. What methodological orientation was stated to underpin the study? e.g. grounded theory, discourse analysis, ethnography, phenomenology | Score a point where the paradigm and methodology are given. Score zero where both aspects are missing. Score U where this information is unclear. | 1 Phenomenology | 1 Interpretive Description methodology | 1 Interpretive Description approach | 1 Interpretive Description | 1 Interpretive Description and inductive analysis approach | 1 Qualitative approach used in-depth, one to one semi-structured interviews | 1 Descriptive design using qualitative research | 1 Hermeneutic Phenomenology | 1 Phenomenology |
| 7.Non-participation How many people refused to participate or dropped out? Reasons? | Score a point where the number and the reason or attempts to identify the reason are given (e.g., a point is score if they say participants would not give a reason for non-participation). Score zero where this information is absent and score u where this information is unclear. | 0 Not mentioned any rejection of participation | 0 Not given | 1 1 became ill & 1 failed to respond | 1 Three participants withdrew because of time conflicts | 0 Not given | 0 Not given | 0 Not given | 1 (1 was admitted to hospital & 1 did not experience any more fatigue) | 0 Not given |
| **Data collection** | | | | | | | | | | |

| | | | | | | | | | | |
|---|---|---|---|---|---|---|---|---|---|---|
| 8. Interview guide Were questions, prompts, guides provided by the authors? Was it pilot tested? | Score a point where testing of the interview script is identified either as a pilot or as a way to determine the content and accuracy of items used. Score a point where consideration to the derivation of questions have come from. | 0<br>Questions guide not pilot tested | 0<br>Not pilot tested but developed without directly mention the concepts of fatigue and exercise to reduce bias. | 1<br>The main question used in previous study | 1 | 1<br>Similar questions used in previous study with women | 1<br>Questions included from a Delphi study | 1 | 1<br>The structure of the interviews was influenced from the quantitative component | 0 |
| 9. Field notes - Were field notes or reflective diary made during and/or after the interview or focus group? | Score a point where field notes are identified. Score zero where field notes are not identified. Score U where this information is unclear. | 0 | 1<br>Immediately after the interview | 1<br>Immediately after the interview | 1<br>During and follow interviews | U | 0 | 0 | 0 | 0 |
| 10. Data saturation Was data saturation discussed? | Score a point where saturation of data is considered. Score a point where another form of sample size reference is made. Score U where this information is unclear. | 1 | 1 | 1 | 1 | 0<br>Not given | 1 | 1 | U | 1 |
| **Total for Domain 2** | | **2/5** | **3/5** | **5/5** | **5/5** | **2/5** | **3/5** | **3/5** | **3/5** | **2/5** |
| | | | | | | | | | | |
| **Domain 3: analysis & findings** | | | | | | | | | | |
| **Data analysis** | | | | | | | | | | |
| 11.Description of the coding tree. Did authors provide a description of the coding tree? | Score a point if an audit trail is given. Score a point if a coding tree is mentioned or score a point if another technique is mentioned that provides a way to structure the information gained. | 1<br>Data triangulation | 1 | 1 | 1 | 1 | 1 | 1 | 1 | 1 |
| 12.Derivation of themes Were themes identified in advance or derived from the data? | Score a point if data driven or theory driven coding is identified or if it is clear how analysis was determined | 1<br>Both | 1<br>Derived from the data | 1<br>Derived from the data | 1<br>Derived from the data | 1<br>Both | 1<br>Derived from the data | 1<br>Derived from the data | 1<br>Both | 1<br>Derived from the data |
| **Reporting** | | | | | | | | | | |

| | | | | | | | | | | |
|---|---|---|---|---|---|---|---|---|---|---|
| 13.Clarity of minor themes Is there a description of diverse cases or discussion of minor themes? | Score a point if there is sub-detail for each major theme. Score no points where this information is absent or u if this information is unclear. | 1 | 1 | 1 | 1 | 1 | 0 | 1 | 0 | 0 |
| **Total for domain 3** | | **3/3** | **3/3** | **3/3** | **3/3** | **3/3** | **2/3** | **3/3** | **2/3** | **2/3** |
| | | | | | | | | | | |
| **Grand Total Score for studies 11/19 – 19/19** | | **8/13** | **8/13** | **9/13** | **9/13** | **6/13** | **8/13** | **6/13** | **8/13** | **8/13** |

## Appendix III Selected type of exercise or intervention's programmes

| **Studies where specified the type of physical activity** | **Type of exercise or intervention's programmes** |
|---|---|
| Clarke, 2015 | Combined strength and aerobic exercise, in the community. |
| Crank, 2017 | Individually tailored supervised exercise comprising supervised and self-directed exercise sessions. |
| Dodd, 2006 | Three exercises on weight machines for the legs (leg press, knee extension, seated heel raise) and three exercises for the arms (lat. pull down, seated chest press, and seated row). |
| Horton, 2015 | Individualized exercise program or exercise of their preference (two participants exercised on their own, one played recreational ice hockey and both went to a gym to work out on their own. |
| Kayes, 2011 | Physical activity included exercise; but also encompassed a range of activities associated with everyday living such as household chores, using the stairs and transportation activity. |
| Learmonth, 2012 | The exercise intervention included differing levels of aerobic, resistance and balance exercises at varying levels of difficulty. |
| Plow, 2009 | A home exercise programme**,** which consisted of stretching, indoor bicycling, and strength training or other types of PA and exercise equivalent to the home exercise programme (e.g. biking outside or going to a gym). |
| Smith, 2009 | A personal tailored exercise programme which included aerobic activities such as static cycling and treadmill walking/ running; strengthening exercises, using body weight resistance and weighted backpacks, whilst performing functional activities such as squats, sit to stands and stair climbing; balance activities, for example tandem stance, single leg stance, foam standing, jumping on a mini-tramp and standing on a wobble board; and flexibility including both participant and physiotherapy facilitated stretches. |
| Smith, 2011 | Community based exercise was defined as a specific exercise activity (e.g. walking, swimming, cycling, gym programme), which took place either at home or in the participants' usual community (e.g. local swimming pool, park, community centre). |
| Smith, 2015 | Community exercises such as walking, cycling, swimming, boxing, using virtual interactive games, or participating in a group exercise class. Community exercise excluded activities in a rehabilitation gym, hospital ward, or other health care setting. |

## Appendix IV C Stage Analysis

| Theme | Sub-theme | Examples | Studies |
|---|---|---|---|
| Exercise | Physical benefit following intervention<br><br>Colours Scheme:<br>Blue: outcomes from activity<br>Purple: perceived benefits from exercise<br>Green & pupil: benefits on fatigue<br>Blue: broader benefits identified<br>Yellow: psychological benefits | Definition of Theme: This sub-theme identifies the perceived benefits and impact exercise had on the physical benefits and functioning following exercise.<br><br>It includes the consequences of these perceived benefits. Across studies, individuals with MS identified perceived improvements from undertaking exercise, including the identification that an individual's strength has improved, or, in the participants own words, 'returned' (6,7,13,16). Individuals identified that their movement, getting around and the achievement of functional tasks, had improved (3,7,11). Other notifiable benefits included increase in the following domains: activity level (1,3), health (3,5), balance (7,9) and flexibility (3,6). Individuals highlighted direct benefits of exercise on fatigue, including reduced levels of fatigue (4,6,13,17), increased endurance (6,12,13), or increased energy (4,9). This was also described as a 'healthy' tiredness (12,13). For instance, one participant stated: 'the fatigue sort of goes into the background' (Janet, I2, P3). (12).<br>These improvements were identified as benefiting the individual's life and ability to undertake activities. Individuals identified an increase in their ability to do activities they could not beforehand, or engaging in new recreation activities, or independence (1,4,5,7,12,17,19). Exercise was identified as a bridge to overcoming barriers to activity (9) and it enabled a sense of achievement (12)<br><br>**VERBATIM STATEMENTS**<br>Most participants had incorporated or increased their levels of incidental exercise (incidental walking, dog walking/training, gardening, playing with children) and/or gentle exercise (walking, yoga) since the intervention. (1)<br>Physical benefits reported by the participants included maintaining and improving health, maintaining muscle tone, improving flexibility, enhancing quality of movement, and increasing activity level. (3)<br>The physical benefits that were most frequently talked about were the feelings of improved fatigue and increased energy and all participants were in agreement about these positive effects. These improvements in fatigue led in turn to increased ability to do things they could not do before. (4)<br>There were new insights into the positive physical, mental, and psychosocial experiences that engaging in an exercise program can bring. "I just really enjoyed it and found it really rewarding" (5)<br>For others, improved health and fitness created new opportunities to engage in recreational activities with their families (5)<br>Not unexpectedly, all nine participants reported that they felt stronger at the end of the programme. Four participants noted improved endurance and two participants noted improved flexibility…. Five participants also reported that various activities of daily living had improved. Seven of the nine participants reported that they had reduced levels of fatigue. (6)<br>The subsequent involvement of this couple in the exercise program has paid substantial dividends, as John believes that Karen's increased physical strength has provided her a degree of independence. In the following quote he describes the | Number of Studies supporting Theme: 1, 3, 4, 5, 6, 7, 9, 11, 12, 13, 16, 17, 19 |

| | | | |
|---|---|---|---|
| | | feelings of guilt he suffered any time he left Karen alone, but how this has eased as her physical functioning improved. (7)<br>I couldn't believe when she started exercising how her strength came back and her balance. (7)<br>I couldn't go out there and sit on a chair without help. But last summer, I did. I was able to. I felt a little bit freer. I'm starting to get a little bit of independence back….(7)<br>When I started (in the exercise program), I could hardly walk from my bedroom to the bathroom and stay up on my feet. I had to struggle to stay on my feet to get from the bed into the bathroom, struggle getting off the toilet, struggle to stand and wash my face and brush my teeth, and now I can do all those things. I'm starting to do some cleaning and tidying. It's just amazing, the difference in my body. (7)<br>Small improvements in physical strength translated into meaningful improvements in day-to-day living. (7)<br>Overall, the exercise class emerged as a bridge to allow participants to overcome barriers to exercise and benefit from the class. (9)<br>One of the main benefits of the class was the improvement of many of the participant's MS symptoms, including balance, mobility/ walking and management of fatigue. Participants felt that exercise helped them to improve their sleeping patterns, activities of daily living and influence healthy lifestyle behaviours. . . . for the last couple of weeks I've been coming up the stairs and down the stairs. (A1) I feel a lot more energetic . . . really I feel much more alert, my head feels clearer I can think things through better. (9)<br>Four of the women reported using physical activity as a form of treatment for their MS, while two other women reported that they engaged in physical activity to lose weight. (11)<br>It [physical activity] does make a difference. I notice I can move better, I know definitely and then you just feel better you know and then I know if I don't do it, I can tell a difference…(11)<br>Furthermore, these perceived physical improvements reduced the amount of mental concentration participants needed to use in order to walk and balance. (12)<br>Perceived physical improvements associated with a healthy tiredness included feeling stronger and lasting longer. (12)<br>Participants felt the experience of a 'healthy' tiredness partially replaced the 'unhealthy' tiredness: 'the fatigue sort of goes into the background' (Janet, I2, P3). A 'healthy' state of tiredness was desired and welcomed, led to a sense of achievement, and induced relaxation and subsequent improvement in sleep quality. (12)<br>Physical improvements included a perceived increase in energy levels, greater strength and endurance, less heaviness in the legs, a 'healthy tiredness', a 'healthy pain' and improved sleep. (13)<br>For example, exercise and physical activity were attributed to improving muscle strength and physical fitness as well as to preventing physical deterioration and loss of mobility. "I do them (exercise and physical activity) to keep mobile really, as much as possible. yeah. it is for mobility really and some of them helps to build up muscles. not big muscles (laughing). (16)<br>Still another said, "I don't know whether it [exercise, working out] has made my MS better or what, but it has increased my stamina where I can do more." (17)<br>Participants identified lifestyle changes as the key modality to managing their fatigue: 'I've actually signed on for yoga' (Fiona). Participants described having made a range of lifestyle and occupational changes after the programme including eating healthier, exercising more, and increasing leisure participation. (19) | |

| | | | |
|---|---|---|---|
| | Psychological Benefits<br><br>Colour scheme:<br>Yellow: psychological<br>Red: social<br>Pupil: spouse feelings<br>Green: general benefits<br>Blue: positive thoughts<br>Dark green: thinking issue | Definition of theme: This sub-theme identifies the perceived psychological benefits following exercise and the positive consequences on participants' quality of life, the way of thinking and their relationships.<br><br>In these studies individuals identified that partaking in physical activities provides a perceived psychological benefit which was expressed as "feeling better" (6,7,11,16). Many of them enjoyed the exercise programme (5,6) and "found it rewarding" (5,6,7,9,11,12,13,16). Two of the exercise outcomes were: a positive perception (4,7,9,12,16) and the improvement in mood or happiness (6,7). Other noticeable psychological benefits were improvement on: stress management (11,12,), empowerment (4,11), confident (1,4,9,11,12) and alertness (9,13).<br>Most of the individuals who participated in community or group exercise highlighted the significance of exercising with people "who were in the same position", which builds good relationship between them, creates fillings of companionship and friendship (4,5,6,7,13,15). The participants perceived group exercise as a strong motivator for "maintaining connections with the world outside of their own home" (15) and encourage them to "do something for themselves" (4,11,16). Some individuals felt the exercise engagement as an "opportunity to challenge their self-limiting thoughts" (9) and enabled them to take control over their symptoms (12,13,16).<br>These perceived benefits encouraged their decision to continue exercising (4,9,11). The perceived physical and physiological improvements provided them a sense of achievement (4,9,12,13,16) which is a significant factor in the process of coping with MS in order to "maintain their identity" (16).<br><br>**VERBATIM STATEMENTS**<br>One key element of the psychological benefits was the group and how the social aspects of group membership contributed strongly to their positive perceptions of the exercise class. They also talked about the "tips and advice" and the support that they gained from each other through exercising with people in similar situations: A4 "for me the social thing was tremendous, meeting people, and meeting people who were in the same position as you." (4)<br>In addition to the group serving to motivate and support them, participants found additional psychological benefits. These included empowerment and confidence and a sense of achievement and pride. (4)<br>They felt that the programme encouraged them to take an active role in managing their illness and enabled them to put the effort into doing something for themselves. (4)<br>… mental, and psychosocial experiences that engaging in an exercise program can bring. "I just really enjoyed it and found it really rewarding" (5)<br>Psychological benefits were described by all nine participants…. An improved mood or happiness was mentioned as a positive outcome by six participants…. Seven participants said they felt enjoyment from participating in the programme. (6)<br>The eight participants who completed the programme valued the social aspect of the programme, including the companionship and friendships that developed. (6)<br>Just as notable as physical benefits was the extent to which exercise positively affected participants' state of mind, and importantly, their perceived quality of life. (7)<br>I just feel guilty when I have a little bit of fun and I don't take her. I don't think I'll ever get rid of that. But it's not too bad now that I know she can get around the house with a walker and cook her own meals and take care of herself. (7) | 4, 6, 7, 9, 11, 12, 13, 16 |

| | | | |
|---|---|---|---|
| | | Clearly, exercise was considered by all of our participants as an important investment in health and wellbeing, but also as a way of maintaining connections with the world outside of their own home. (7)<br>Spouses were also mindful of the social aspect of exercise, and the potential of keeping their partners engaged in the outside world. (7)<br>Participants were questioned on whether they felt that they had achieved any personal goals; some participants felt that they had done so. My confidence is getting better as well, and my balance is getting better. The goals I set myself . . . I'm achieving some of them. (A1) Others felt that with continued exercise they could improve more. We could all do better yet. (9)<br>…the class gave them the confidence and opportunity to exercise again. Several participants had been active in the past, but were not active now; the class gave them the opportunity to challenge their self-limiting thoughts,… (9)<br>. . really I feel much more alert, my head feels clearer I can think things through better. (9)<br>I'm just getting some physio and some exercises you know to try and gain my strength….I feel good that I've done that for myself like you know it, it's psychologically good for me…(11)<br>One can see that physical activity is used for more than just the physical benefits, but also to improve their psychological health by providing an outlet for stress management and to improve self-esteem and self-efficacy. (11)<br>I know definitely and then you just feel better you know and then I know if I don't do it, I can tell a difference…(11)<br>Furthermore, these perceived physical improvements reduced the amount of mental concentration participants needed to use in order to walk and balance. This in turn led to participants experiencing less mental tiredness. 'Rachel' explained this: I'm not sort of consciously worrying what my right foot is doing as much which is good. …I wasn't sort of worrying about what was going on and that was a good feeling. I think I was more confident in myself (Rachel I2, P1). Positive feelings associated with healthy tiredness included a sense of achievement, learning to listen to the body, taking control and improved confidence…… Increased feelings of relaxation, achievement and confidence in turn also led to a better quality of sleep. (12)<br>Psychological improvements included a sense of achievement, an improved sense of control, greater relaxation and a higher degree of alertness. (13) One participant described with enthusiasm the positive nature of a group with which she exercises on a weekly basis. (13)<br>…participants felt both exercise and physical activity gave them a 'feel good factor' and sense of achievement. feel good factor. …I don't know what it is in your body that when you exercise it sort of seems to release all these bits and pieces and it makes you feel better. (16)<br>In summary, exercise and physical activity was a way of coping with the condition. However, participants described the strategies used not only for coping with MS but also in maintaining their identity, which symbolised much more than having a diagnosis with MS. Participants desired to be known for their individuality; physical activity was therefore used as a way to shape and preserve their sense of self. (16) | |
| | Limited levels<br><br>Colour scheme: | Definition of theme: This sub-theme identifies the perceived restrictions to exercise, the consequences of vigorous exercise and the necessary modifications to become bearable to them. It also includes negative post-exercise outcomes of some participants who decided to stop exercising. | 1, 5, 6, 7, 10, 12 |

| | | | |
|---|---|---|---|
| | Red: difficulty to meet activity recommendation<br>Yellow: suitable exercise<br>Green: since diagnosis get inactive<br>Blue: exercise aggravate/ provoke symptoms<br>Pupil: psychological consequences<br>Dark red: stop activity because fatigue | Across studies many of MS individuals perceived a reduction of their physical capacity after diagnosis (5,7) with negative physical and psychological consequences (5,7). For instance, one participant reported that was unable to make a step without help and felt as a prisoner in her house (7). Although many of them could not meet the recommended level of activities (1), they did suitable adaptations (1,5) or coped with their musculoskeletal problems (6) in order to remain active.<br>However, some of the individuals who perceived worsening of fatigue related symptoms preferred to stop exercising (5,10,12).<br><br>**VERBATIM STATEMENTS**<br>But while most participants reported engaging in some form of exercise, they largely did not meet the recommended lifestyle intervention (1)<br>Others said that despite understanding the recommendations, incidental exercise was the best they could do: "I probably fall short of [the recommendation] because it is meant to be vigorous. I wouldn't do three times a week of vigorous exercise, but I get plenty of incidental exercise." (P6.3, 39 y, mild). (1)<br>However, a diagnosis of MS had created a transition toward physical inactivity and a perceived reduction in exercise capacity for many participants: "I would say I became very inactive" (FG1:2). Other participants attempted to maintain their exercise levels but found it difficult: "I was still a member of a gym, but it ended up that I would drive to the gym and then I would be sat in tears in the car thinking I can't do that. so I gave up going to the gym because it is supposed to be there to help me but it was carving me up and making my symptoms worse" …(5)<br>One participant reported finding the exercises difficult and at times experiencing some pain, but was still able to complete the program, achieving a volume of exercise that was manageable for her. (5)<br>Five participants reported musculoskeletal problems such as aches and pains, but each participant without prompting from the interviewer particularly noted that these problems were not directly related to or affected their ability to complete the programme. The only musculoskeletal problem that resulted in missed sessions was experienced by Participant 7: 'I ended up with a severely painful back . . . I missed several sessions . . . but the pain has now gone. (6)<br>The other musculoskeletal problem was muscle soreness during and after the sessions. This was reported by three participants. These muscle problems were generally experienced in the first week of the 10-week programme…(6)<br>I felt I was a prisoner in my own home the last year, before I started this program. I had anger issues, I had frustration issues. I would stand at the window and look out and think 'I want to just walk down my driveway and get the mail'. You can't even step outside without help. (7)<br>For example, participant #13 did not like going to the wellness group sessions because of her fatigue…(10)<br>Feelings of shakiness and heaviness in the legs were occasionally experienced by some participants. Sarah described this: 'There have been a couple of days I felt really tired . . . I have a wee bit of trouble walking then and sometimes my left leg can sort of drag a wee bit . . . I just feel I can't do anymore.' (Sarah I3, P4) (12) | |
| Barrier to Exercise | Tiredness<br><br>Colour scheme:<br>Red: fatigue | Definition of theme: This sub-theme identifies the perceived increase of fatigue during or after physical activity, mostly when it is combined with full-time working and dealing with housework and/or family care. This sense of fatigue restricts exercise motivation and participation, either because the patients could not cope with it, or by the fear of exacerbation. | 1, 2, 3, 6, 7, 8, 9, 10, 12, 13, 15 |

| | | | |
|---|---|---|---|
| | Yellow: reason of fatigue<br>Green: impact to exercise<br>Blue: copping<br>Pupil: emotional response | It includes the negative emotional response caused by fatigue and overlapping strategies. Across the studies participants indicated a severe sense of tiredness which was worsening while coping with everyday life commitments in general (1,2,7,8,15), during or post-exercise (3,6,7,8,9,12,13,15) and exercising over "the edge" [post-exertional (9,12)]. This sense of fatigue may also be related to environmental issues, as is the surrounding noise and/or when other people move close to them during the activity (13), the conditions during the preparation for exercising (dressing/ undressing, walking to reach the equipment (10), or it may be depended on the previous night's sleep (15), if patients exercise without rest (7) and combination with other activities (2,7,9). Most of the participants reported unable to deal with this unhealthy tiredness (12) and preferred to stop their engagement with physical activity (1,2,3,7,8,9,10,12,13,15), even though some of them knew that the symptoms of fatigue do not last long (6,8) and after the fist difficult period, exercise might increase their energy levels. These individuals could not potentially muster the necessary energy to overlap this difficult stage (8). For instance, a participant stated that: "It's overwhelming. It's the way I'd say, you just, you don't really care about anything but wanting to have a rest" (8).<br>Not all individuals perceived this sense of fatigue as an incomparable factor (3,6,9) and those were able to monitor their fatigue levels were empowered to continue enjoying their physical activity (3,9). However, the vast majority of the participants who experienced high levels of fatigue reported unable to exercise and some of them felt that exercise is a waste of time (15), it did not meet their expectations (12), or it was worsening their symptoms (13). For example, a participant described this sense: "you're back to square one [to the beginning]."(15). This negative emotional response was accompanied by feelings of failure and anxiety and hindered exercise participation (15).<br><br>**<u>VERBATIM STATEMENTS</u>**<br>Others said fatigue played a role too: "It was all a bit much, working full-time, dealing with the house. I don't like to say fatigue, but I would get tired and there just didn't seem to be enough hours in the day to do everything" (P6.1, 55 y, mild). (1)<br>A number of participants reported suffering extreme fatigue during exercise, or from coping with life in general which acted as a barrier to exercise. The physical and cognitive energy costs of fatigue hindered their exercise motivation and participation. (2)<br>Exacerbation of fatigue was the only reported negative physical effect of AF classes. Participants discussed the importance of monitoring their fatigue levels while in the pool so that they could continue to enjoy the program. …<br>while high levels of fatigue had a negative impact on participation (3)<br>Increased feelings of fatigue were reported by one participant. Participant 9 stated that he 'was alright immediately afterwards but then the following day . . . I wasn't physically outgoing or as strong, I probably needed to rest more'. He went on to say when asked how long this had lasted 'Oh very short . . . the following day I was fine again'.(6)<br>On certain days, with the increased amount of fatigue associated with MS, I think it's very difficult to get up and get motivated to get going and do it, and not to feel so tired that the rest of your day is lost because you put out a certain amount of energy to do a bit of exercise. (7)<br>Fatigue is a fairly big symptom and I do get tired if I do too much at once. (7) | |

| | | | |
|---|---|---|---|
| | | …when their initial experience of fatigue impacted on their ability to engage in physical activity in the first place. They could see that taking part in physical activity would help increase their energy levels, but they just could not muster the energy to get going in the first place. (8)<br>…the experience of fatigue was itself perceived to be a barrier to taking part in physical activity by the majority of participants. For example, some participants tried to describe their experience of fatigue and its impact on their ability to take part in physical activity. 'It's overwhelming. It's the way I'd say, you just, you don't really care about anything but wanting to have a rest. (8)<br>…only being able to participate in physical activity if the effort required to initiate engagement in the activity was not too great, because if they expended too much energy at the outset, they rarely had enough energy left to actually take part in the physical activity. (8)<br>'I used to go to the MS swimming thing at Papatoetoe . . . but then I was finding I was too tired on Thursday then, after doing all that. I mean, it was quite hard work what we did for an hour in the pool, so I'd find that I was too tired on a Thursday, which was harming my work. Which is more. . .? I feel it is more important carrying on in a work place, so. . .' (Hannah, Age 39, Secondary progressive MS) (8)<br>However, for some participants' post-exertional fatigue immediately after the class was seen as a negative outcome. My fatigue's got worse, but my balance has improved. (9)<br>Post-exertional fatigue, previously described in the "benefits of the class" theme, was discussed by some participants as being problematic. This could be seen as a potential barrier to exercise, although not all participants deemed post-exertional fatigue problematic. (9)<br>For example, participant #4 reported, 'I have tried to workout at a gym, but by the time you park your car, change your clothes, and fight your way to the equipment, I am too tired to exercise'. (10)<br>Sometimes active and inactive participants described how symptoms, such as fatigue, were worsened after engaging in PA and perceived that symptoms were often too severe to engage in PA. For example, participant #13 thought that using the mini-cycle may have caused her exacerbation. (10)<br>Participant #5 had a goal of exercising three times a week because she felt it would help reduce fatigue. However, she felt that some days her fatigue was so severe that she could not exercise. (10)<br>In participants with little or developing control, recognition of 'the edge' was not always consistent during exercise sessions and going over 'the edge' was associated with an unhealthy tiredness. (12)<br>In addition, negative feelings accompanied unhealthy tiredness and included feelings of failure, anxiety and inability to cope with exercise: 'It just means I come home and have a rest, so it doesn't matter but it doesn't make you feel quite so good about the session that you've meet the expectations' (Em I3, P5) (12)<br>Pam explains this: The things that make me tired don't make somebody else tired and things I can do I can't necessarily do in a busy environment or where there's a lot of movement, noise or background noise or people moving in front of me. (13)<br>At the follow-up interview, one participant had stopped exercising due to increasing levels of tiredness and a perceived worsening of her MS. (13)<br>"If I take the dog for a walk, I pick out how far I'm going to go, . . . and some days it's a cool day and you can go down there and I can walk quite well for 500 meters . . . [but] other days, if you haven't had a good sleep, then you can walk a | |

| | | | |
|---|---|---|---|
| | | 100 meters. He goes on to say that "some days I think, you know, it's a waste of time because I'd rather just go and sit on the couch, because after exercise, "you're back to square one [to the beginning]." (15) | |
| | Anxiety of pushing themselves to the limit<br><br>Colour scheme:<br>Red: patient reports<br>Yellow: negative emotions<br>Pupil: beliefs<br>Blue: physical effects<br>Green: happy medium (fair balance) | Definition of theme: This sub-theme identifies the participants' perceived fear of over-pressing themselves to the limit. There is a general consensus that there is a "fine line" between beneficial and damaging exercise, so if someone crosses this line they might experience adverse effects.<br><br>Across the studies participants reported that they were cautious about not over exerting themselves (2,3,8,11,13) and some of them stated that were conscious about their limits (8,11,13) while others generally avoided pushing themselves too hard (2,3,11). This cautious behaviour is based on the belief that MS individuals should keep a fair balance on physical activity (8,11,13) in order to experience the exercise benefits without having adverse symptoms. Participants who cross the "fine line" up to where physical activity is beneficial, experienced a worsening of fatigue related symptoms (2,3,5,8,11,13) which could last for several days or cause them to relapse (11,13). For instance, a participant stated: "I never want to overexert myself with exercise at all because I'm afraid it will bring it on…my thinking now is do what you can do and that's all you can do. I think you have to be careful…because you are in danger of compromising the other aspects of your quality of life…you just have to find that proper balance… it's such a no brainer to me" (11). The perceived consequences of the over exertion are physical [loss of physical capacity, increase of fatigue which affects their coping ability (2,5,12,13)] and emotional [affects their mood, makes them feel negative, inadequate (12,13)]. Participants were frustrated when some people pressed upon them to do more (13) without recognising their limits because they were afraid of the harmful consequences of over exertion (2,3,5,8,11,). However, health care professionals contended that "context and experience" of MS individuals influence the potential benefit or harm of exercise (14).<br><br>**VERBATIM STATEMENTS**<br>He was also cautious about over exerting himself for the fear of losing even more physical capacity. (2)<br>I don't push myself to the limit, if I push myself to the limit, yes I would feel the fatigue more and it would have an adverse effect...(2)<br>One participant explained, "You can push yourself too hard and then you don't want to go back. It's not fun anymore." (3)<br>I gave up going to the gym because it is supposed to be there to help me, but it was carving me up and making my symptoms worse " (5)<br>This belief relates to the idea that there is a fine line between exercise that is beneficial and exercise that is damaging (8)<br>However, this belief in a 'fine line' between benefit and harm, adds a caveat to this that physical activity is beneficial, so long as you keep within a certain threshold and if one crosses this threshold, then the consequences could be detrimental. 'I've discovered that there's a line between exercise that is beneficial and exercise that isn't. You know that you've gone over the line of doing more damage than good for me. (8)<br>That last relapse was in October 2006… I think it was after I did the breast cancer run because I really overexerted myself. I never want to overexert myself with exercise at all because I'm afraid it will bring it on…my thinking now is do what you can do and that's all you can do. I think you have to be careful…because you are in danger of | 2, 3, 5, 8, 11, 12, 13, 14 |

| | | | |
|---|---|---|---|
| | | compromising the other aspects of your quality of life…you just have to find that proper balance… it's such a no brainer to me. (11)<br>Most of the participants described being able to control their symptoms by avoiding physical exertion, since this exertion often worsened their symptoms or caused them to relapse. (11)<br>'Well it's because I got to the stage when I was feeling so tired I wasn't really, I don't think I was following the instructions clearly, and that just makes you feel inadequate I think . . . I just think it makes you feel worse about the situation, you don't feel so positive about it' (Em. I3, P1). (12)<br>Most participants strictly adhered to their limits, as the consequences of overdoing it were perceived as extremely negative and could affect areas such as mood and coping ability for several days. (13)<br>People often try to push people to do more . . . and it's like I know my limits and its quite frustrating that to them it's like what's the difference going to be? But there's a big difference and I think it's a fine line. (13)<br>Exercise as a concept also created a dilemma and was considered by several participants as potentially beneficial or harmful depending on context and experience of the individual. (14) | |
| | Importance of exercise<br><br>Colour scheme:<br>Green: difficult with exercise<br>Blue available to exercise<br>Red: Exercise activity with no effect<br>Yellow barriers to exercise<br>Pupil: How use<br>Dark blue: exercise beneficial | Definition of theme: This sub-theme identifies the perceived benefit from physical activity as the main motivation in exercise prioritisation for MS individuals' daily schedule. Negative beliefs related to the efficiency of exercising prevent the MS individuals' from spending any effort to continue being active. In contrast, individuals who perceive the benefit of exercise put it in high priority into their daily schedule.<br><br>Across the studies individuals who believe that exercise has no effect on the disease progress (2,8,10) or consider that exercise demands high effort without any significant effect in improving their physical health (1,2,7,8,13,16), consequently choose to remain inactive (1,2,7,8,13,16). For instance, one participant stated: 'It's like having a tin can with holes punched in it, no matter how much water you pour into it, it is still pouring out" (8). A second group of participants identifies the benefits of exercise in improving their general (1,10,14,16,17) or mental (11) health and focus on their difficulties in prioritising it into their daily programme (2,7,13,14,16). Some of them were reluctant to alter or differently prioritise their interests (1,7,16), while others consider more beneficial to input a few exercises into their daily activities than to follow a formal exercise programme (17).<br>There is one last category of participants who they recognise the importance of regular exercise for maintaining their strength (1,11,16), feel blessed to be able to put it into their schedule (1,16) and perceive the satisfaction of doing something for themselves (11).<br><br>**VERBATIM STATEMENTS**<br>One participant compared adhering to the different recommendations and said: "So I think (the diet) is easier because we have to eat, and with the exercise and the other things there, you think, well… yeah, I know I'm a bit complacent with the exercise and meditation" (P1.1, 37 y, mild). (1)<br>On the other hand, participants who were retired or working part-time were more easily able to incorporate exercise into their schedules: "I find that most days [exercise] fits into place. I'm fortunate, because I'm retired and I haven't got any working commitments or anything like that." (P1.2, 65 y, mod). (1) | 1, 2, 7, 8, 10, 11, 13, 14, 16, 17 |

| | | | |
|---|---|---|---|
| | | I have the feeling that if I'm going to use my limited energy, I might as well use it productively to do some job I want to do or to take part in some activity rather than just to use it (my energy), go to a gym and do the activity to no effect. I don't have a lot of spare energy, so I use it to good effect than to no effect. (2)<br>There was a sense of 'opportunity cost', in that energy is a limited resource that had to be conserved. Participants felt that the energy and effort that is required to get through an exercise session has been spent and is therefore not available for other activities. (7)<br>People think, 'if I have limited energy to do something, should I use that energy to go to the gym to work out or should I use that energy to pick my kid up after school and walk them to the park?'(7)<br>The belief that physical activity is a waste of time seemed related to previous post-MS experiences of physical activity and was at least in part linked to the degenerative nature of the illness, in that the benefits of physical activity were not always evident due to disease progression. 'It's like having a tin can with holes punched in it, no matter how much water you pour into it, it is still pouring out. (8)<br>… many had mixed feelings on how well exercise helped them to manage symptoms and/or prevent MS from progressing. Participant #2 said: 'As far as the disease itself is concerned, I'm completely indifferent [to the effects of exercise], but exercise always benefits me, its exercise.' Participant #13 felt that exercise was beneficial in improving mental health, but not beneficial in improving physical health. (10)<br>I'm just getting some physio and some exercises you know to try and gain my strength….I feel good that I've done that for myself…(11)<br>This participant felt she could be more economical with her energy if exercises could be incorporated into activities of daily living, such as household chores, as she could not see a stand-alone exercise programme helping her with functional difficulties. (13)<br>… these factors were attributed to the personal interests, priorities, and positive or negative attitudes of the individual client. (14)<br>Jill expressed that her priorities were in order of family, home and everyday physical activity. After these activities were completed the remaining energy was attributed to exercise. my focus is on getting all the stuff I have to get done, done for the family and then any extra energy I can use on physio. it's extremely urm frustrating to get to the end of the day and think, I just haven't got the energy. (16)<br>….so earlier when they (children) were in school. I would like to visit the parents evening and things like that so then that would be prioritised. I would be prioritising those things. So exercise just keeps going down. (16)<br>One participant talked about the benefit of throwing a few exercises into one's daily activities rather than trying to establish and maintain a formal exercise program. (17) | |
| | Lack of appropriate knowledge/ understanding of exercise providers | Definition of theme: This sub-theme identifies the lack of knowledge of health care professionals and exercise instructors and also a gap of communication between professionals and clients. It describes the difficulties MS individuals experienced and how these influenced their physical capacity.<br><br>Across all these studies, participants highlighted their need to have access to a knowledgeable professional/ instructor to give them an informed exercise advice (4,5,12), to device a programme (2,4,5,7) and give them a feedback to be sure they perform the exercises properly (4). However, most of them agreed (2,4,5,7,9,12,14) that professional exercise advice was: | 2, 4, 5, 7, 9, 12, 13, 14,19 |

| | | | |
|---|---|---|---|
| | Colour scheme:<br>Red: what patient need from whom<br>Yellow: what should do<br>Green: what (believe) has done<br>Blue: lack of knowledge<br>Pupil: consequences | "either non-existent or not relevant for the needs of PwMS" (5). For instance, a participant described his experience: "My physiotherapist referred me to the local sports centre, and then a gentleman was trying to make me a plan of what to do, and he hadn't dealt with anybody with MS before, and actually it was a bit of a disaster" (5). Health care professionals postulated that the lack of scientific knowledge regarding the physiological nature of fatigue and the invisible character of this impairment are responsible for the inappropriate fatigue interventions (5,7,9,12,1314) and a personal experience approach for fatigue management based on their own "tried and tested" (12) behaviour (14). This inadequate exercise advice lead MS individuals to believe that they know more than their instructors about what is suitable for them, so they choose to exercise independently (2,14) or they prefer to stop exercising (9). Another participant explained his previous experience: "I felt that the individual trainers … they didn't really have an understanding, they were frightened as well … they were sort of saying well you can't do this and you can't do that. I would be like, oh well, I won't go at all." (9). Participants are more reluctant to exercise with professionals who adopt negative approaches as: "one size fits all", "sympathy and pity" (13), when the professionals always win in their one-to-one interaction (19), or when they perceive that "health care provider help is only available for those with a visible or progressing disability" (13).<br><br>**<u>VERBATIM STATEMENTS</u>**<br>The gym themselves, they do have people who work there that can help with exercise programs,….I'm sure if I had to I could sit down with them and devise a programme but I know more than they do about what is suitable. (2)<br>They talked about the importance of a knowledgeable instructor who could advise how much or what to do when and about the need for the instructor to give feedback to ensure they were doing the exercises properly…(4)<br>There was <u>general consensus</u> that exercise advice from health professionals was either non-existent or not relevant for the needs of PwMS. (5)<br>"My physiotherapist referred me to the local sports center, and then a gentleman was trying to make me a plan of what to do, and he hadn't dealt with anybody with MS before, and actually it was a bit of a disaster".(5)<br>I was told not to". Rob mentioned the cautious nature of those advising him in the early stages. I don't think the neurologist was all for it at first for any of us, to tell you the truth. When you go there, you do see the physiotherapist and she gives you a lot of bending and stretching exercises…. I did those for years and they did help a little but that was it. (7)<br>…the negative attitude and lack of knowledge of health and leisure professionals, which had resulted in <u>participants not exercising in the past</u>. . I felt that the individual trainers [in previous exercise experiences], although they were very sympathetic, they didn't really have an understanding, they were frightened as well . . . they were sort of saying well you can't do this and you can't do that. I would be like, oh well, I won't go at all. (9)<br>''Mitch'' described an exercise session where the therapist encouraged him to listen to his body: 'I don't think he tried to control me as much. He let me <u>play with the buttons</u> and increase the speed and everything. (12)<br>Aspects of health care provider encounters that were perceived negatively included: a 'one size fits all' approach, sympathy and pity, advice or instruction that the participant perceived to be counterintuitive and the message <u>that health care provider help was only available for those with a visible or progressing disability</u>. (13)<br>…people with MS-related fatigue were more likely to <u>seek exercise activities independently</u> rather than through an HCP as a result of invisible impairments. (14) | |

| | | | |
|---|---|---|---|
| | | Participants also felt that a lack of knowledge existed between healthcare team members with regard to their role within the interdisciplinary team. (14)<br>A perceived dearth of knowledge regarding the physiological nature of fatigue and interventions for fatigue was problematic for study participants. In the absence of scientific knowledge, participants in this study discussed how unproven theories and "gurus" might influence practice. Some participants relied more on personal experience when approaching fatigue management and offered exercise and nutrition guidelines based on their own "tried and tested" health behaviours. (14)<br>…you know all my interactions with professionals have been on one-to-one up to this and um . . . obviously when you are dealing with the physiotherapy, when you strongly believe in something um . . . you know it's your view against theirs and they are the professionals, so they are most likely to win. (19) | |
| | Lack of motivation and support<br><br>Colour scheme:<br>Red: importance of support<br>Blue: barriers to exercise<br>Yellow: effects of lack of support<br>Pupil: potential reason of PwMS behaviour | Definition of theme: This sub-theme identifies the perceived need for motivation and support by health care professionals, family and friends in order to be engaged in a regular exercise programme.<br><br>Across the studies participants recognised the significance of professional and/or family support during exercise programme to exercise with consistency (4,5,7). MS individuals complained that physicians and NHS rehabilitation centres did not provide enough support and opportunities to maintain activity "in the way of managing their disease and its accompanying symptoms." (9,11). Patients who participated in group therapy expressed their difficulties to remain motivated without the professional support, after the end of the exercise programme (5,9). For instance, a participant stated that: "I've had the incentive to come, and I've enjoyed coming. Doing it at home and now it's finished I've just gone back to how it was before" (5). Some of the participants reported the influence of insufficient understanding from friends and family on their perceived support (13) and how in some cases family and friends' behaviour act as barrier for them to be active (10). In contrast, spouses praised their role to keeping involved partners in exercise (7). Health care professionals postulated that the lack of interest and motivation in exercise "were attributed to personality, motivational, and cognitive changes associated with the process of MS" (14).<br><br>**VERBATIM STATEMENTS**<br>They were finding that without the support and discipline of the once a week classes they were finding it much more difficult to keep exercising: A2 "I kind of miss that to be honest, that motivation" and C3 "You really have to dig deep to motivate yourself to do them[the exercises]."(4)<br>"I've found that because you have no structure once you are at home, you do tend to sit around and watch a bit more TV rather than when you have to come [to the center] when you're at home, you think I'll not bother today" (FG6:3), and "I've had the incentive to come, and I've enjoyed coming. Doing it at home and now it's finished I've just gone back to how it was before" (5)<br>At the same time, the spouses were cognizant of the important role they themselves could play in terms of facilitating their spouses' involvement in exercise and helping to overcome isolation. (7)<br>Participants commented on the lack of opportunities to exercise….[The NHS rehabilitation centre] couldn't obviously do that for us (group exercise). This is a wee outlet for us all, and I think it would be good for us all if it continued. (9) | 4, 5, 7, 9, 10, 11, 13, 14 |

| | | | |
|---|---|---|---|
| | | While most participants had a story of how the social environment interfered with PA, sometimes active and inactive participants more often cited family and friends as barriers to PA (10)<br>Many felt that their physicians did not provide them with much support in the way of managing their disease and its accompanying symptoms. (11)<br>Lack of understanding from friends and family led to a dearth of perceived support in several participants. (13)<br>Lack of motivation and lack of interest in exercise were attributed to two different sources in people with MS…… these same factors were attributed to personality, motivational, and cognitive changes associated with the process of MS. (14) | |
| | Busyness or other interests preventing exercise<br>Colour scheme:<br>Green: commitments/ interests<br>Red: reason<br>Pupil: Make/ find time<br>Yellow: not available to exercise<br>Brown: other obstacle: heat | Definition of theme: This sub-theme identifies the perceived personal commitments which compete the exercise and the main reasons imposing stop or limit exercising.<br><br>Across the studies individuals stated that work (1,2,8), family (1,10, 14,16) and other personal or social (10) responsibilities require most of their daily time (1) or energy (2,8,10,16). Participants who prioritise their role of carrying for work or family as more important than their own health (14), could not take part in regular exercise activities (1,2,8,10), or chose to spend only their remaining energy to exercise (16). For example, one of the participants although recognised the need to find or make time to exercise, he stated: "it's just not available to me. I want to do it, it's just juggling life, getting that life balance." (1).<br><br>**<u>VERBATIM STATEMENTS</u>**<br>Participants described the "busyness" of their day-to-day routines—working, running errands, looking after children and family, and consequently feeling as if they were leading very active lives. (1)<br>Most participants reported an episodic pattern of exercise, with a range of barriers and competing interests that prevented a regular regimen (1)<br>By far, the perceived time commitment required to fulfil the recommendation was the most difficult obstacle to regular, vigorous exercise. Competing interests on time were mentioned by almost all participants. Participants who were employed (especially in full-time employment) and/or had family and caring commitments talked about needing to find or make the time in their day to exercise, and often this was not possible: "I think ideally I would like to be doing [exercise] all the time, but I'm realistic in that at the age and phase of my life… I don't have a lot of that time I can put into exercise and meditating or whatever else because it's just not available to me. I want to do it, it's just juggling life, getting that life balance." (P4.2, 46 y, mild). (1)<br>I have the feeling that if I'm going to use my limited energy, I might as well use it productively to do some job I want to do or to take part in some activity… (2)<br>'I used to go to the MS swimming thing at Papatoetoe . . . but then I was finding I was too tired on Thursday then, after doing all that. I mean, it was quite hard work what we did for an hour in the pool, so I'd find that I was too tired on a Thursday, which was harming my work. Which is more. . .? I feel it is more important carrying on in a work place, so. . .' (Hannah, Age 39, Secondary progressive MS) (8)<br>Participant #6 described how other social activities interfered with exercise: '[My friends] want to do social stuff sometimes. "Oh let's get a coffee". You know, and I'm really likely to just give up and drink coffee'. (10) | 1, 2, 8, 10, 12, 14, 16 |

| | | | |
|---|---|---|---|
| | | Participant #11 described how a family crisis led to her non-adherence after a period of exercising: 'I finally got myself up to that [exercising five days a week] before my brother called. I get this phone call [saying my brother had cancer] and everything had changed'. (10)<br>But because the stress level was high, the responsibility was more. Not just because of the heat but because of everything, the stress, just the whole, all of it built into one summer. I was like ehhh; you know the summer from Hades made it difficult to exercise. (10)<br>All three inactive participants reported considerable stress in their life. For example, participant #11 felt guilty about not exercising, but also felt that coping with her brother having cancer, changing jobs, and recovering from an exacerbation made exercise too difficult. Participant #12 frequently had to take care of her grandchild, which she believed increased her stress and fatigue. (10)<br>… a client prioritized his or her role caring for family, yet the health professional felt the client should prioritize his or her own health. (14)<br>Jill expressed that her priorities were in order of family, home and everyday physical activity. After these activities were completed the remaining energy was attributed to exercise. (16) | |
| | Deterioration in functioning or increase in symptoms<br><br>Colour scheme:<br>Green: deterioration symptoms<br>Red: affected activities<br>Blue: cognitive & emotional effects<br>Yellow: poor exercise outcomes | Definition of theme: This sub-theme identifies the perceived physical decline which was experienced by individuals with MS after a variety of physical activities and their negative physical and cognitive consequences on them. It includes their influence on participants' choices to stop exercising selectively or totally<br><br>In these studies some individuals mentioned a perceived functional decline (1,9), or limited energy (2), or lack of balance (2), or raised body temperature (5,18), or pins and needles on legs (5), or injuries (1) as a consequence of their engagement in episodic (1) or structured exercise (1,2,9,18) or even in simple daily activities (2).<br>The symptoms were experienced during and/or after the activity, had negative cognitive and emotional impact on them and led them to avoid exercising (1,2,5,8,9,18). For example, a participant stated: "I wasn't able to do any of that anymore, well I just kind of stopped doing anything" (9).<br><br>**VERBATIM STATEMENTS**<br>Participants with functional decline or fear of falls reported having difficulty with finding a safe way of doing vigorous exercise: "I was doing aqua aerobics and chair-based yoga as well, but I had to stop because I deteriorated" (P3.1, 57 y, sev) (1)<br>The participants engaging in episodic exercise also described having experienced injuries, MS related symptoms, or illness. (1)<br>I have the feeling that if I'm going to use my limited energy, I might as well use it productively to do some job I want to do or to take part in some activity…Nathan's loss of functional capacity clearly limited his ability to do every day physical activity or structured exercise. (2)<br>I don't risk climbing ladders anymore because of my balance. (2)<br>Lesley spoke of how balance affected her exercise experience, but experienced physical problems which resulted in a negative cognitive experience: I have problems with balance which is one of the reasons why I found swimming difficult because I felt unsteady, unsupported in the water and anxious that I was going to do a nosedive. (2) | 1, 2, 5, 8, 9, 18 |

| | | | |
|---|---|---|---|
| | | "You are scared because you immediately get the symptoms from the increased body temperature and everything anyway, my feet automatically have pins and needles all up my legs and that is murder… (5)<br>The experience of taking part in physical activity with seemingly no positive impact was linked to an emotional response where participants began to experience a sense of helplessness or frustration with physical activity in the absence of a positive response. 'It feels good at the time, but when you try to do the same thing again the next day you only get a quarter of the way back, that's depressing'. (8)<br>Pre MS, I was fairly fit . . . I did do a programme of exercise, a lot of swimming and sit-ups to build the muscles up. But MS kicked in and I wasn't able to do any of that anymore, well I just kind of stopped doing anything. (9)<br>Participants often cited raised body temperature as a barrier to exercising for endurance, as this added to fatigue. (18) | |
| | Lack of patients' information<br><br>Colour scheme:<br>Red: lack of…<br>Green: effects lack of | Definition of theme: This sub-theme identifies the lack of patients' knowledge about the appropriate exercises for them, how these might affect their symptoms and the potential results.<br><br>Across the studies, MS individuals highlighted the lack of information from health care professionals after their diagnosis, regarding exercise (1,4,5,7,8,), lifestyle advice (5) and relevant services available from the community and leisure industries (14). This lack of knowledge related to exercise perpetuates old beliefs and attitudes (7). Patients before their engagement to the programme of exercise still wondered if exercise is beneficial or might do "more damage than good" (4,7,8).<br>Participants indicated the significance of being informed about the exercise programmes, the benefits and the possible consequences (7,8), in order to avoid injuries due to inappropriate exercise (1) and to interpret and "understand the symptoms they are experiencing" (8).<br><br>**VERBATIM STATEMENTS**<br>The participants engaging in episodic exercise also described having experienced injuries, MS related symptoms, or illness. (1)<br>The majority of participants spoke about their limited knowledge before the programme of exercise for PwMS; they were afraid exercise might make their condition worse. Participants described a shift from thinking that exercise might do harm to knowing it was beneficial: A2 said "I'd be kind of worried about doing more damage than good, when I wouldn't know how to use the equipment or something like that"…(4)<br>Participants recalled that exercise and lifestyle advice was not generally offered after their MS diagnosis: "I don't recall any professionals mentioning anything about exercise at all, and I don't believe I asked" (5)<br>Old attitudes occasionally prevail, in the sense that exercise is still regarded as somewhat of a risky endeavour. (7)<br>…the lack of information about the benefits of physical activity and what one may expect as a result of physical activity may lead people to interpret the symptoms they experience after physical activity as a sign that they are making their MS worse. (8)<br>Information about what one can expect to experience as a result of physical activity may help people to understand the symptoms they are experiencing. (8)<br>...participants described a lack of knowledge in people with MS of "what's out there" regarding services and assistance available both in the healthcare as well as the community and leisure industries. (14) | 1,4, 5, 7, 8, 14 |

| | | | |
|---|---|---|---|
| | Geographical distance<br><br>Colour scheme:<br>Yellow: barriers<br>Red: Solutions<br>Blue: facilitations<br>Pupil: stop activity | Definition of theme: This sub-theme identifies the perceived barriers on MS individuals to access athletic facilities. Geographical distance, lack of disable parking and difficulties to use various means of transportation are some of the mentioned barriers which finally prevent some from partaking in regular exercise. It includes the facilities which enable some of them to continue exercising.<br><br>Across the studies participants indicated some of their objective difficulties to approach the nearest athletic facility. Long geographical distance (1), lack of disabled parking (8) and transportation difficulties (14,16) are some of the mentioned barriers which demand high energy consumption to approach the facilities (1,8,10,14) and consequently restrict their ability to exercise (1,9,10,16). One of the participants clearly depicted: "If I had to fight for a car park and then walk to it after I had been swimming, I just probably wouldn't do it. It's like . . . what do I want to use my energy for? Do I want to use my energy for a battle to find a car park, or do I want to use my energy to swim and exercise my body really well?" (8)<br>Individuals who could easily reach the facilities or to use the disable parking spaces had no disturbances to their physical activities (1,8).<br><br>**<u>VERBATIM STATEMENTS</u>**<br>Geographical distance from facilities was another barrier to exercise: "I could go to a gym, which would be a 25-kilometre drive well, I suppose I could, but I just don't." (P7.1, 51 y, mild). Conversely, one participant stated: "the exercise one is getting easier as we go along, especially since I moved into the retirement village and the facilities are there for me to use" (P5.3, 63 y, mod). (1)<br>'I honestly don't think I would go swimming if I didn't have that [a disability card]. It means that I know I am going to get a park right outside the swimming pool. If I had to fight for a car park and then walk to it after I had been swimming, I just probably wouldn't do it. It's like . . . what do I want to use my energy for? Do I want to use my energy for a battle to find a car park, or do I want to use my energy to swim and exercise my body really well?' (8)<br>… others commented on the lack of disabled parking spaces or mentioned that transport could be problematic. This information provided an insight into the external barriers which could prevent people who were moderately affected with MS from taking part in exercise classes. (9)<br>Some reported that the swimming pool was too warm or that the facility was not easily accessible, and that getting there increased their fatigue. (10)<br>Financial limitations and transport difficulties were also discussed by participants…(14)<br>Mary described difficulties associated with navigating different modes of transportation resulting in her reducing her physical activity and doing more driving in order to sustain her important activities. (16) | 1, 8, 9, 10, 14, 16 |
| | Weather<br><br>Colour scheme:<br>Green: weather conditions | Definition of theme: This sub-theme identifies the perceived weather-related barriers and their consequences on MS individuals' symptoms which cause a temporary discontinuation of weather dependant activities.<br><br>Across the studies many participants reported exaggeration of fatigue-related symptoms and lack of energy in hot weather (1,10,13,1517), including direct and intense sunlight as well. It has been stated that humidity influences the energy levels and makes the muscles stiffer (17). One participant stated that: "[The humidity] kills me. It saps whatever | 1, 10, 13, 15, 17,18 |

| | | | |
|---|---|---|---|
| | Pupil: provoked symptoms<br>Red: stopped activities<br>Blue: emotional & cognitive symptoms<br>Yellow: possible solution | energy is there to begin with. It makes my muscles feel heavier. It makes me feel sluggish, groggy... It is one of the few things that I can count on as creating some cognitive symptoms as well" (17). In cold climates, low temperature causes stiffness and pain, affects balance and may influence the energy levels (13,15,17), while wind inhibits some participants to walk outside because they feel unsafe (13). Most of the participants expressed their difficulty to continue exercising in extreme outdoors temperature (1,10,13,15).<br>Air-conditioned gym could be a potential solution for heat barrier (18) but some of the participants reported difficulty exercising even in an air-conditioned room with extreme heat outside (10).<br><br>**VERBATIM STATEMENTS**<br>Inclement weather was noted as impacting on motivation, with weather-dependant activities more likely to be paused or discontinued during winter months, especially in colder climates. (1)<br>The study took place over the summer. Thus, hot weather, which exacerbates fatigue [43], was frequently cited as a PA barrier. Participants in all three groups reported difficulty exercising, even indoors, when it was hot outside. Participant #1 said, 'It was very hard to persevere with the extreme heat. I lacked energy and therefore motivation'. Participant #7 reported that exercising, even in an air-conditioned room, such as a gym, was difficult when the weather was hot. (10)<br>Not just because of the heat but because of everything, the stress, just the whole, all of it built into one summer. I was like ehhh; you know the summer from Hades made it difficult to exercise. (10)<br>On the other hand, participant #6 recognised that she used her anxiety about possibly exacerbating her fatigue in hot weather, as an excuse not to exercise. (10)<br>Environment seemed to play an important part in activity choice for several participants in this study. Overheating affected some participants' energy levels, balance ability and vision. (13)<br>In contrast, several participants described how colder weather affected the physical symptoms of MS such as balance, and physical sensations such as stiffness and pain. One participant described the wind as a barrier to walking outdoors as she felt less safe. (13)<br>Several men described how extremes of temperature, particularly heat, could influence fatigue levels and subsequently limit the amount of exercise they could complete. Most men referred to environmental heat and sunlight as key triggers for fatigue; however, others described how extreme cold also could influence energy levels. (15)<br>Participants noted that their fatigue increased related to a number of environmental factors, including heat; direct, intense sunlight; humidity; and cold. (17)<br>[The humidity] kills me. It saps whatever energy is there to begin with. It makes my muscles feel heavier. It makes me feel sluggish, groggy... It is one of the few things that I can count on as creating some cognitive symptoms as well. (17)<br>Although air-conditioned gymnasia could be promoted as a way of dealing with this problem, their cost often was identified as a barrier. (18) | |
| | Anxiety for the future<br><br>Colour scheme: | Definition of theme: This sub-theme identifies the perceived anxiety for the future and a potential exaggeration of MS symptoms and how this sense inhibits physical activities and aspirations.<br><br>In these studies, individuals who feel a permanent (2) or a temporary loss of control on their body as a result of a worsening fatigue (8,12,13), or they do not know the type of MS and the potential illness progress (16), might experience | 2, 8, 12, 13, 16 |

| | | | |
|---|---|---|---|
| | Green: MS symptoms<br>Red: Anxiety for future<br>Blue: wonder for worsening of symptoms | a sense of uncertainty about their ability to perform both present and future physical activities (2,12). This negative emotion influences their future plans, choices and aspirations (12). When depression interweaved with anxiety for the future may lead individuals to think that any effort for improvement is in vein (13). For example, Angela stated: 'everything goes to pot' (13).<br><br>**VERBATIM STATEMENTS**<br>I'm more restricted, I now take care before I do something, I think 'Can I do it?' 'Am I capable of doing it?' 'How I am going to be afterwards? (2)<br>In emotional response, if previous experiences of physical activity had resulted in a worsening of fatigue, they often resulted in anxiety and a fear of participating in physical activity in the future. (8)<br>…an overwhelming sense of fatigue with controlling characteristics which influenced both present activities (such as cooking a meal or performing well at work) and future aspirations (such as planning a career, considering children's futures, buying a house and going on holiday).(12)<br>For example, Angela described a feeling of depression, which involved a fear for the future, and led to not being able to think about normal things: 'everything goes to pot' including deterioration in balance, walking and increased heaviness in the legs. (13)<br>For example, the uncertainty about the type of MS and its implications for the future. I don't know how mine is going to progress. I don't even know what type of MS I've got. urm. (16) | |
| | Dependence on others<br><br>Colour scheme:<br>Red: perceived limitations<br>Green: need help<br>Pupil: barrier to exercise<br>Blue: emotional response<br>Dark red: increase dependency | Definition of theme: This sub-theme identifies the perceived physical limitations and the growing need for help from others which shatter their independency<br><br>Across the studies, participants expressed the perceived dependency for performing simple daily activities (2,7,13,15,18) and to participate in physical activities (2,15) as well. Claire, one of the participants, emphasised her reliance on others for partaking in regular exercise:" I think it would help if I had somebody to go with, someone that could help me physically as well as sort of going in…" (2). MS participants felt vulnerable (2), severe limited and unable to control their life (2,7) because of loss of body control (2,7) and their spouse felt guilty when left their partner unsupported (7). For instance, a participant stated: "I felt I was a prisoner in my own home the last year, before I started this program. I had anger issues, I had frustration issues. I would stand at the window and look out and think 'I want to just walk down my driveway and get the mail'. You can't even step outside without help" (7).<br>Participants described their tendency to avoid asking for help from others, because this increases their perceived dependency (13,15,18) and they allowed themselves to ask for help mostly from close family members, only when they had no other option (15,18). In addition, they delegated an activity to someone else for preventing experienced fatigue and ensuring that the task will have been completed (18). MS individuals prefer not to use mobility devices in order to maintain their independency (13,18) and physical capacity (18) as they avoid being visually different (18) to other people.<br><br>**VERBATIM STATEMENTS**<br>The limitations of not being in control of your own body and life, somebody else has to do everything for you…(2) | 2, 7, 13, 15, 18 |

| | | | |
|---|---|---|---|
| | | Some of the participants felt vulnerable and required support from others when exercising. Claire emphasised this point: I think it would help if I had somebody to go with, someone that could help me physically as well as sort of going in…(2)<br>In the following quote he describes the feelings of guilt he suffered any time he left Karen alone, but how this has eased as her physical functioning improved. I couldn't leave her and I had no one to come and stay with her. I felt guilty going and leaving her, getting out and doing something and not being able to take her.(7)<br>I felt I was a prisoner in my own home the last year, before I started this program. I had anger issues, I had frustration issues. I would stand at the window and look out and think 'I want to just walk down my driveway and get the mail'. You can't even step outside without help. (7)<br>Fear of weight gain featured in participant descriptions: one participant strongly associated <u>weight gain</u> with reliance on a <u>wheelchair</u>, and another with <u>growing dependency</u> on others. (13)<br>As a result of this perceived dependency, he was unwilling to ask his wife to drive him to exercise classes in the city. (15)<br>In general, people did not like asking for help, often declaring that they valued their independence. P5 (U.S female) explained: Sometimes, I guess, I ask someone to help when I don't know what else to do, and there seems like there's no other option,…. (18)<br>Participants commonly associated using a mobility device with loss of independence and strength, which they wanted to maintain, or not wanting to be different. (18)<br>Ask someone for help was the strategy most frequently identified in this category (64.5%). Although participants indicated they asked close family members such as their spouse/partner or children for help, often they appeared to be more reluctant to ask others outside close family for assistance. In general, people did not like asking for help, often declaring that they valued their independence. P5 (18)<br>Delegate an activity to someone else in the 'Used Sometimes' category (51.6%). The advantage of using this strategy was that it would prevent them from becoming fatigued while ensuring that the task was completed. (18) | |
| | Fear<br><br>Colour scheme:<br>Green: types of fear<br>Pupil: physical limitations<br>Blue: stopped activities because of fear<br>Red: past negative experiences | Definition of theme: This sub-theme identifies the perceived fear of the consequences of a fall and/or injury which inhibits MS individuals to participate in formal exercise. It includes the reasoning of that negative emotional response.<br><br>Across the studies, it is evident that individuals who have a level of functional decline (1,12) or they had a past negative experience of falling and/or injury, might experience a sense of fear, mostly in an environment which was perceived as less safe (1,13). For instance, a participant stated: "Going to the gym was just too hard and too treacherous. Too many opportunities to trip and fall." (P5.1, 55 y, sev) (1). This negative response linked to the belief that a potential injury will have more severe consequences for them than for a healthy person, so they try to prevent this possibility by avoiding any "risky" activity (1,8,12) and selecting "alternative forms of exercise over which they experience more perceived control" (13).<br><br>**<u>VERBATIM STATEMENTS</u>**<br>Participants with functional decline or fear of falls reported I have trouble with one of my legs… Walking is just not doing it, but I don't think I could run without falling over." (P6.1, 55 y, mild). (1) | 1, 8, 12, 13 |

| | | | |
|---|---|---|---|
| | Yellow: positive management | "Going to the gym was just too hard and too treacherous. Too many opportunities to trip and fall." (P5.1, 55 y, sev). (1)<br>Another commonly referenced emotional response was fear, in particular a fear of falling and/or a fear of injury. Many of those who had fallen or experienced an injury previously suggested that they had stopped taking part in physical activity, or certain activities, due to a fear that they would fall or injure themselves again. 'For me, having the experience I had when I sprained my ankle, I am just aware that if I get injured the consequences for me are going to be a lot more serious than they are for somebody else . . . potentially. So, I just try to avoid situations where I am going to get injured really'. (Cheryl, Age 41, Relapsing remitting MS) (8)<br>Participants felt that walking and balance were subsequently perceived to be affected by an unhealthy tiredness, leading some participants to feel unsafe following an exercise session. (12)<br>Several participants expressed fear of falling, particularly whilst walking outside, and this frequently led to alternative forms of exercise over which they experienced more perceived control. (13) | |
| | Conflicting recommendation<br><br>Colour scheme:<br>Red: conflicting advice<br>Yellow: their effects | Definition of theme: This sub-theme identifies the ambiguity of health care providers regarding exercise advice and its effects on patients' motivation.<br><br>Across the studies participants mentioned the lack of exercise advice from some group of health care providers (5,7,8,14). For instance, Cheryl stated: "I don't think the medical profession promotes exercise. You know . . . whether they don't see it as their job... I mean I like my neurologist... she is very good, but she would never have spoken to me about exercise and neither really did the MS nurse at the hospital". (Cheryl, Age 41, Relapsing remitting MS) (8). MS individuals highlighted their need to receive definite recommendations about exercise, the nature of fatigue and its management in order to partake in exercise in regular basis (7).<br>Health care professionals described how physiotherapists and occupational therapists have a different approach in regard to fatigue management and this "interprofessional conflict" influences negatively the patients (14). In addition, the complex nature of fatigue leads professionals from different boundaries to conflict regarding the scope and ownership of the suggesting intervention with result "some participants perceived their roles to be undervalued and, at times, poorly understood or undermined by other members of the healthcare team" (14).<br><br>**VERBATIM STATEMENTS**<br>Participants felt that endorsement from the clinical team was needed to confirm that exercise was something you were allowed to and supposed to do, but this advice was variable: "When I was diagnosed I asked specifically if there was anything I could do to help myself, diet wise and exercise, and I was told there was nothing you could do whatsoever" (5)<br>The historical ambivalence of some health professionals towards exercise due to concerns related to fatigue and heat sensitivity issues magnified the general reluctance expressed by people with MS to partake in physical activity. (7)<br>'I don't think the medical profession promotes exercise. You know . . . whether they don't see it as their job . . . I mean I like my neurologist . . . she is very good, but she would never have spoken to me about exercise and neither really did the MS nurse at the hospital'. (Cheryl, Age 41, Relapsing remitting MS) (8) | 5, 7,8, 14 |

| | | | |
|---|---|---|---|
| | | Several areas of conflict were described by participants in this study, including interprofessional conflict, provider versus client conflict, knowledge conflicts, and client motivational conflicts. The perceived nature of fatigue led to interprofessional conflict regarding "ownership" of fatigue, particularly in PT and OT participants in the study. Subsequently, this resulted in further conflict in regard to fatigue management approache.… Interprofessional conflict was perceived by HCP participants as having negative influences on clients. (14)<br><br>Blurring of professional boundaries seemed to be related to the complex nature of MS-related fatigue, changes in roles over time, and conflicts regarding perceived "ownership" of roles. Some participants felt their roles were changing; for example, one participant explained that the role of exercise had changed from maintenance of existing movement and functional status to focusing on improving cardiovascular function, muscle strength, quality of life, and long-term good health. Some participants perceived their roles to be undervalued and, at times, poorly understood or undermined by other members of the healthcare team. (14) | |
| | Comparison with healthy people<br><br>Colour scheme:<br>Red: barrier to exercising in public activities<br>Blue: mental & emotion outcomes<br>Green: difficulties to cope<br>Pupil: Potentials copping strategies | Definition of theme: This sub-theme identifies the difficulty experienced by MS individuals exercising publicly and their negative emotions when comparing themselves to healthy people.<br>It includes some of the coping methods that were used to address it. Across the studies MS individuals mentioned that exercising in a public environment causes an obvious comparison between them and healthy people. Although these differences are not always in appearance, MS individuals feel different (9,13,14,15) or disabled (13), so they feel reluctant to exercise publicly (9,13,14,15). Participants reported embarrassed to explain their physical limitations [lack of stamina (13), weakness & lack of balance (15)] to their social peers and expressed their worries that members of the public perceive them as intoxicated (13). In addition, this sense of difference influences them to feel negative emotionally. For instance, a participant stated that: "sometimes it's quite, when you see super fit people, it's a wee bit... (A4); Depressing, maybe?" (9).<br>C. Smith (15) highlighted that men show a preference to exercise alone to avoid being embarrassed and to be able to manage easier the duration of their activity or to include some rests. Women (13) feel more comfortable exercising in groups and implement other coping methods. A woman participant reported that: "attended a class with older people who she felt were 'wiser or something…" (13).<br><br>**<u>VERBATIM STATEMENTS</u>**<br>We're all the same, there is nobody any different, which was a bonus, because sometimes it's quite, when you see super fit people, it's a wee bit. . . (A4); Depressing, maybe? (9)<br>Participants discussed that exercising in an environment with healthy people was also a barrier for them. Participants often felt different from healthy people and that they had to explain their illness or their symptoms after activity. (9)<br>Several participants described particular embarrassment at being thought of as intoxicated by members of the public. (13)<br>Some felt that exercise was promoted for mainstream people' but not enough for people with disabilities. One participant described the difficulties in explaining to her social peers her progressive loss of stamina, and attended a class with older people who she felt were 'wiser or something… (13) | 9, 13, 14, 15 |

| | | | |
|---|---|---|---|
| | | …the invisible nature of fatigue often led to clients feeling as if they were "different," even though these differences were not outwardly apparent. These feelings resulted in a reluctance to exercise publicly, for example, in a gym or at a swimming pool. (14)<br>Several men chose to exercise alone rather than go to a gym or a group exercise setting because they could stop and rest easily or shorten exercise duration without being embarrassed in a public place.<br>Choice of exercise setting enabled the men to self-pace activities, which seemed more important for those whose fatigue resulted in weakness and balance difficulties. Preferred exercise settings differed between men in this study and women in a previous study (13) who appeared to be more comfortable exercising in a group. (15) | |
| | Financial limitation<br><br>No Colour scheme | Definition of theme: This subtheme identifies the cost required by MS individuals for exercising in a gym, class, pool or with a trainer as a barrier for maintaining active.<br>Across the studies participants reported their need to cover the additional cost of a regular exercise on a weekly base in a professional environment with appropriate equipment (1,14,18) and also that financial limitations is a barrier for exercising (18).<br><br>**<u>VERBATIM STATEMENTS</u>**<br>Participants did note that maintaining regular exercise could be resource-intensive. Classes, gym and pool passes, exercise equipment, and professional and/or clinical consultation were noted to all incur a cost. One participant stated: "You need to have a bit of finances." (P6.2, 49 y, mild) about the resources required for swimming (squad) training. (1)<br>Financial limitations and transport difficulties were also discussed by participants…(14)<br>Although air-conditioned gymnasia could be promoted as a way of dealing with this problem, their cost often was identified as a barrier. (18) | 1, 14, 18 |
| Fatigue related consequences | Negative feelings related to fatigue<br><br>Colour scheme:<br>Red: loss of…<br>Yellow: due to…<br>Blue: negative emotional response<br>Green: physical deficiency<br>Pupil: descriptions | Definition of theme: This sub-theme identifies the perceived effects of fatigue physically and emotionally. It includes many narrative descriptions where participants expressed how they experienced fatigue on their body and their entire life.<br><br>Across the studies participants expressed how fatigue caused a sense of heaviness in the muscles, the head and the whole body (8,15), unsteadiness on their limps (16), a sense of emptiness when the energy gone (15,17), and triggers deteriorating balance (15). Participants' narratives explained their individual experiences and the perceived effects of fatigue: "Wearing a trench coat that goes down to your ankles and it's made of lead." (15), "I feel as though I have got concrete blocks. That is how I feel all the time." (Donna,8), "After exercise you feel sort of emptied out and your…body's sort of empty." (15), "…it literally feels like someone cut off my left arm..., and just feels like the blood and all the energy that I have in my body is kind of going out at the same time." (17). It is obvious that every individual experienced differently the physical effects of fatigue (14), which might cause the loss or reduction of many meaningful and/or pleasurable physical activities (2,7,8,10,12,13,14,15,16,17). In addition, the inability of some individuals to adjust their goal with the proceeded physical deficit (15) combined with the disengagement from highly valued activities (15) such as work, driving, exercising, social relationships (2,15,16) might result in a sense of permanent loss of things (8,16). For instance, a participant described it as: "Your body just shuts you down" (8), while Ivy articulated her experience since she gave up painting: "…as you get worse, you lose, urm... different abilities to do things. And you sort of, especially | 2, 7, 8, 10, 12, 13, 14, 15, 16, 17 |

with my art, you sort of go through a temporary, what do they call it? A temporary sort of grieving time. It's a loss" (16). These participants perceived fatigue as a threat to their self-identity (15), as the hardest symptom of MS to manage (12) and believed that it had complete control over them (12,15,17). This belief was often followed by negative feelings including fear, anxiety, anger, sadness, depression, and expressing helplessness over disease (8,10,12,13). Unfortunately, some participants after some stressful events adopted negative coping styles, such as being in denial about MS (10) and refused to do anything or to go anywhere (7).

However, Katherine described how the physical effects of fatigue depended on depression levels: "Well, it tends to ... my frame of mind as well, you know if I can sink into quite deep depression as well and when I feel really low I tend to not have a lot of energy, and then other days I can be feeling really good and yeah I feel sort of happy and full of energy" (Katherine, I1, P1). (12)

**<u>VERBATIM STATEMENTS</u>**

The narratives clearly reflected the loss of exercise enjoyment due to reduced ability, and profound loss of skill to be active…(2)

Nathan describes how he missed not being able to be as active…(2)

The loss to social connectedness through sport was one of the consequences of MS, coupled with loss of exercise enjoyments like 'runner's high' and not being outdoors. (2)

He didn't feel well with the fatigue, it hurt to move, he didn't want to do anything, he didn't want to go anywhere. So there was both the emotional and physical aspects of everything that was happening to him. (7)

I was just out of it, gone, wasted. Your body just shuts you down'. (8)

'It's like you have a piece of elastic, you know, knicker elastic, you know how it perishes? It will keep stretching back to its original shape if you only stretch it a small way, like about two thirds of its stretch, but if you decide to stretch it all the way it never goes back, that's what I mean'. (8)

In emotional response, if previous experiences of physical activity had resulted in a worsening of fatigue, they often resulted in anxiety and a fear of participating in physical activity in the future. (8)

'Really heavy. . .. I feel as though I have got concrete blocks. That is how I feel all the time'. (Donna, Age 52, Relapsing remitting MS) (8)

In addition to these stressful events, participant #11, #12, and #13 displayed negative dispositional coping styles, such as being in denial about MS, avoiding MS-related functions, and expressing helplessness over the disease. (10)

Katherine explains: 'Well, it tends to . . . my frame of mind as well, you know if I can sink into quite deep depression as well and when I feel really low I tend to not have a lot of energy, and then other days I can be feeling really good and yeah I feel sort of happy and full of energy' (Katherine, I1, P1). (12)

Participants spoke of many negative feelings including fear, anxiety, anger, sadness and guilt and these participants believed <u>fatigue was their biggest problem and the hardest symptom</u> of MS to deal with,… the fatigue does, the fatigue is the thing that has complete control over me and that's just absolute power. I can't do anything about it, it controls me, it would control my day if it could'' (12)

Angela described the fluctuating and unpredictable changes in fatigue as the 'nature of the beast', which appeared to reflect the negativity experienced by all participants with regards to MS-related fatigue. (13)

| | | | |
|---|---|---|---|
| | | …the ways in which fatigue was experienced by clients varied both between and within individuals… If nobody knows what fatigue is you can quite understand why there's a lot of differing beliefs about it . . . . (Participant 2) (14)<br>The men in this study experienced physical fatigue in the muscles, head, and entire body. Whole-body experiences were likened to "wearing a trench coat that goes down to your ankles and it's made of lead." (15)<br>"After exercise you feel sort of emptied out and your . . .body's sort of empty." (15)<br>…if fatigue triggered deteriorating balance, men often described embarrassment about appearing drunk and were worried about how others identified them. (15)<br>Many, but not all, of the men in this study described how fatigue posed a threat to their self-identity in the form of progressive losses that included meaningful work, youth, the authority to drive, strength and energy, and relationship roles. Such losses caused one man to say he felt "like I'm old before my time." (15)<br>Conversely, **those who are unable to successfully make goal adjustments** can **disengage from highly valued activities**, **resulting in negative feelings**. (15)<br>…"the depression response is most likely to occur when the frustrated goal is deeply connected to the core of the self. Such a goal is not easily abandoned or even scaled back" (p. 1721). (15)<br>The loss of meaningful physical activity was described as akin to the grieving process. This was most clearly articulated by Ivy who could no longer continue her art due to physical limitations imposed by her MS. it's almost two years … I've had to give up painting because I'm right handed but my right hand isn't steady enough. I'm not a loose painter of water colour. and I'm not going to start after all these years, trying to be a loose painter, I'd go mad,. as you get worse, you lose, urm. different abilities to do things. and you sort of, especially with my art, you sort of go through a temporary, what do they call it? A temporary sort of grieving time. it's a loss. (16)<br>Another said, " . . . you never feel like you did in the old days before you had this deep fatigue, and there is no way for anybody else to measure that except to know that you know you never feel like you did before." (17)<br>"Fatigue just rules my life or tries to." (17)<br>When talking about fatigue-related emotional changes, participants talked about crying, screaming at the kids, irritability, and "optimism kind of goes down." One individual said, "When it is not at work, but at home, and I am just emotionally exhausted, I get irritable pretty easily. (17)<br>." Another related, "I almost start to cry because my concentration goes." And yet another, "I can't remember anything, and I don't want to remember anything." (17)<br>"I have had a lot of experiences where what it feels like is I just got hit by a tidal wave.., kind of feeling like I am in the undertow and I can't get out." Another related " . . . it literally feels like someone cut off my left arm.., and just feels like the blood and all the energy that I have in my body is kind of going out at the same time." (17) | |
| | Reduction of bodily control<br><br>Colour scheme:<br>Red: restricted activity | Definition of theme: This sub-theme identifies some of the perceived physical and psychological consequences of fatigue, its specific nature and how it affects MS individuals' life.<br><br>Across the studies participants described a progressive reduction of their mobility, associated with MS (13,16) and a sudden physical deterioration, which might be predictable [for example: "I have tried to run, I can do about half a mile, good half mile and then the legs do start to become weak after running for half a mile and my feet start to trail a little…" (2)] or unpredictable (8,14,17). Participants highlighted that unpredictability of fatigue combined with the invisibility and | 2, 8, 12, 13, 14, 15, 16, 17 |

| | | | |
|---|---|---|---|
| | Yellow: physical consequences<br>Blue: psychological consequences<br>Green: Fatigue character | individuality (14) is a major problem and consequently affects their self-integrity (13) and alters their life (14). For instance, a participant stated: "Because in a mall, in stores, there is no place to sit down, and I'd just--I have no—you know, when you are that tired, you just have no pride; you are just going to sit down on the floor underneath the clothes rack. I mean, I didn't care, and I guess that is probably an example; I didn't care who saw me or where I was. I just didn't care. I was exhausted" (17).<br>Participants reported some of the physical effects of fatigue: lack of energy (8), weakness (2), deterioration (12), tripping (2), heaviness (13), loss of balance (17), increased body temperature (17), knees buckle (17), reduction of reflexes in driving (17). These temporary physical symptoms might be accompanied by "emotional irritability and cognitive difficulties as impaired memory, attention, and concentration" which are often related to high fatigue levels (17). Several MS individuals were able to recognise the pre-fatigue symptoms such as the onset of head pressure (13), increasing heaviness in the leg (12,13) and an increased tendency to trip (2,13). These sensations enable them to prevent the worst fatigue symptoms (13). However, one of the participants expressed a different option and stated that: "I will go for a walk on the treadmill... and my legs will be all jellylike, but after a few minutes—10, 15 minutes—you recover again" (15).<br><br>**VERBATIM STATEMENTS**<br>I struggle walking upstairs, I can walk up one flight of stairs, anything more than that's a struggle, so if I've got an option I'll take the lift. (2)<br>Well, on the few times I have tried to run, I can do about half a mile, good half mile and then the legs do start to become weak after running for half a mile and my feet start to trail a little, I have a job to, I can't pick them up, I'm in danger of tripping (2)<br>This is made more complex by the fact that MS is unpredictable in nature as this participant went on to describe: 'But the problem is that the line doesn't stay stationary. It's the same with most MS people. What you did yesterday is not necessarily what you could do today, but you might be able to do twice what you did the day before tomorrow, it's very fluid, that level is always fluid'.(8)<br>….you can use up all that energy that's available on the day in one foul burst . . . and then, you know . . . like I've done once before . . . I did two or three days of a lot of things with the girls in the school holidays, so we were very busy. We did two or three days no problem and on the fourth day I was just out of it, gone, wasted. (8)<br>…perceived physical deterioration and negative feelings may increase the fatigue experience in people with MS. (12)<br>Several participants described loss of control, which they associated with both MS and fatigue. This loss of control appeared to threaten the self-integrity of the individual. (13)<br>These limits were expressed by the body in several ways. Several participants used altered physical sensation in order to recognise limits; these sensations included the onset of head pressure, and signs of muscle fatigue such as increasing heaviness in the leg and an increased tendency to trip. (13)<br>Difficulties presented by fatigue included characteristics of unpredictability, invisibility, and individuality and was consequently life altering for people with MS. (14)<br>One man's description of muscle fatigue following exercise was typical of most participants: "I will go for a walk on the treadmill . . . and my legs will be all jellylike, but after a few minutes—10, 15 minutes—you recover again." (15) | |

| | | | |
|---|---|---|---|
| | | These changes over time had an impact on her daily activities: it has changed because… I could pop down the road to get something from the shops. but I can't now. so, I can't just walk down the road! (16)<br>A temporary increase in physical symptoms (e.g., weakness, loss of balance), emotional irritability, and cognitive difficulties (impaired memory, attention, and concentration) was frequently associated with informant's descriptions of the effects of increasing levels of fatigue. (17)<br>…"when you are fatigued just everything goes wrong. You lose your balance . . . . Everything gets worse," " . . . my legs just get real fired and quit . . . . my knees buckle," and "I don't trust my reflexes when I am tired with my driving." (17)<br>….to illustrate the very whelming effect of fatigue and her experience of the paralyzing force:<br>" . . . I would just literally sit down on the floor in a section while they're doing their stuff. Because in a mall, in stores, there is no place to sit down, and I'd just--I have no—you know, when you are that tired, you just have no pride; you are just going to sit down on the floor underneath the clothes rack. I mean, I didn't care, and I guess that is probably an example; I didn't care who saw me or where I was. I just didn't care. I was exhausted!" (17) | |
| | Imposed daily planning<br><br>Colour scheme:<br>Red: be planed<br>Pupil: negative consequences of planning<br>Green: causes of planning<br>Blue: desirable activities<br>Yellow: emotional response<br>Dark blue: important parameters | Definition of theme: This sub-theme identifies the necessity of planning and organizing the required activities in order to overcome the limitations of perceived fatigue.<br><br>It includes the need for prioritising activities in place of others on a daily or weekly base and the consequences of this strategy. Across the studies the most frequently used fatigue related coping strategy (83.8% in 18) is planning the daily (7,11,16,18) or weekly (8,18) activities. Participants described that everything should be planned (2,7,8,11,13,16,18), even the simplest daily activities, to have the necessary energy to complete them. MS individuals mentioned their attempt to predict the duration of their time-limited energy (11,16,18) in correspondence with a desirable activity (7,8,16,18). However, the fluctuating nature of fatigue (13) makes the success of their plans unpredictable with consequent negative emotional responses "were often described as frustrating and limiting" (13). For example, a participant stated: "Mainly, it's the worry about the fatigue, if I overdo it, I get the fatigue and you know, sort of balancing the two... I balance my life around it, you know" (8). Other negative consequences of planning were mentioned, like loss of the opportunity to be impulsive (2), to act spontaneously (2,13,18), to be dependable (2) and also that it makes every simple activity seems like expedition (16). A significant parameter of the planning is the prioritisation (8,16,18) which is used when imposed by the circumstances (18) [the majority of MS individuals might do one think every day (8,16)], where participants should determine what is important to do and choose some tasks over others (8,16,18).<br><br>**VERBATIM STATEMENTS**<br>Everything has got to be planned. Every single thing. You can't be impulsive as you'd like but literally everything needs to be planned. The loss of spontaneity, dependability and the clear need to plan for every movement when living with MS is powerfully expressed within these narratives. (2)<br>The energy required simply to manage everyday activities is often significant, and thereby affects what else can be attempted in a day. (7)<br>'Mainly, it's the worry about the fatigue, if I overdo it, I get the fatigue and you know, sort of balancing the two. . .. I balance my life around it, you know. We had a party Saturday night . . . I rested most of Saturday afternoon, so that I could enjoy the evening'. (8) | 2, 7, 8, 11, 13, 16, 18 |

| | | | |
|---|---|---|---|
| | | I have to plan my day. If I'm going to the mall then I decide what stores I'm gonna go to, 'cause I know that I'm only good for such and such amount of time and so I have to plan you know what I'm doing…(11)<br>The fluctuating and unpredictable nature of fatigue dictated continual adjustment through pacing and planning, strategies which themselves were often described as frustrating and limiting, and led to a loss of spontaneity in life. (13)<br>Everything is a major planning, expedition really if I'm going out! (16)<br>As such, priorities became overwhelmingly influenced by their personal choice as well as their energy demands." every day with this illness, because you're aware that you've got this limited amount of energy if you like… I just plan one activity, if I've got to go to the shops, I just have one visit and the rest of the day I'll collapse in a heap and you do one thing each day…well that's how you have to organise your life now, everything in your brain, what you do when you wake up is all organised around fatigue and the time you've got to do things in even dressing. (16)<br>By planning and organizing, people attempted to avoid situations where their fatigue prevented them from participating in or continuing an activity. (18)<br>Plan and organize the day was the strategy most frequently identified as being used regularly (83.8%).…planning sufficient time to complete an activity was emphasized as important. By planning, people attempted to prevent 'being caught' in a position where they might be fatigued and could not use other strategies to manage it. (18)<br>…planning and organizing their week allowed them to conserve energy on some days in order to have it available on other days. (18)<br>The disadvantage identified of planning was that it removed the opportunity to act spontaneously. (18)<br>**Choose not to do an activity in order to do something else** also was placed in the 'Sometimes' 'category by 16 people (51.6%). Participants linked this strategy to determining what was important to them and prioritizing some tasks over others. Its use depended on circumstances. (18) | |
| | Cognitive effects<br><br>Colour scheme:<br>Red: cognitive impairments<br>Dark red: physical impairments<br>Yellow: Emotional effects<br>Green: Fatigue characteristics<br>Blue: consequences | Definition of theme: This sub-theme identifies the perceived cognitive symptoms related to fatigue which might affect MS individuals after physical or mental tiredness.<br><br>Participants described the reduction of their cognitive capacity (2,10,13,14,15,17) which develops the following symptoms: limited memory (10,17), lack of concentration (13,17), lack of attention (17), "loss of judgment and/or perception" (17) a mental fogginess (13), "brain-cheese", a "hazy, out-of-body fatigue feeling," "a hangover" (15), "feel like being in a dream", "feel sluggish" and "feel groggy" (17). MS individuals experienced these symptoms isolated or combined with the physical sense of fatigue which might be associated with emotional irritability and depression (13,14,17). Participants described them as "different states of fatigue" (13). Some of them experienced more often one than another, or occasionally one state of fatigue might trigger another (13).<br>Mentally working participants expressed difficulties to concentrate and participate in meetings, while others described their willing to shortening their ongoing conversations (17). For instance, one participant stated: "... it is harder for me to follow a train of thought; it's harder for me to respond appropriately. It is harder to stay engage" (17).<br><br>**VERBATIM STATEMENTS**<br>MS fatigue that makes coping with MS much harder, and it has serious implications for physical and cognitive capacity. (2) | 2, 10, 13, 14, 15, 17 |

| | | | |
|---|---|---|---|
| | | Participant #13, talking about her cognitive impairment, said: 'the ability is limited, the ability to remember or not remember [to exercise] is problematic'. (10)<br>In addition, physical and cognitive symptoms, such as poor balance and concentration, were also discussed in the context of fatigue experiences. (13)<br>Participants described different states of fatigue including muscle weakness, a mental fogginess, feelings of depression and an all-consuming fatigue. Furthermore, many participants had experienced more than one state of fatigue during the course of their MS and, on occasion, one state of fatigue could trigger another. (13)<br>MS-related fatigue was perceived as having very little scientific explanation and often seemed inextricably linked to other MS-related impairments such as depression, poor balance, and cognitive difficulties. (14)<br>Head experiences were described as "brain-cheese," a "hazy, out-of-body fatigue feeling," and a hangover." (15)<br>A temporary increase in physical symptoms (e.g., weakness, loss of balance), emotional irritability, and cognitive difficulties (impaired memory, attention, and concentration) was frequently associated with informant's descriptions of the effects of increasing levels of fatigue. (17)<br>Cognitive changes were characterized as; "like being in a dream," "inability to concentrate," and "loss of judgment and/or perception." Several working participants talked about difficulties related to concentrating and participating in meetings. One said, " . . . it is harder for me to follow a train of thought; it's harder for me to respond appropriately. It is harder to stay engaged." Another said, "Talking with people, you don't want the conversation to go very long, just to finish as soon as possible (17)<br>[The humidity] kills me. It saps whatever energy is there to begin with. It makes my muscles feel heavier. It makes me feel sluggish, groggy... It is one of the few things that I can count on as creating some cognitive symptoms as well. (17) | |
| | Effects on Employment<br><br>Colour scheme:<br>Green: cause<br>Red: unable to work<br>Light rose fired<br>Dark rose: choose to reduce commitments<br>Blue: emotional impact<br>Yellow: positive copping | Definition of theme: This sub-theme identifies the undesirable loss of working capacity as a consequence of fatigue and its physical and emotional response on MS individuals' life.<br><br>Across the studies participants reported the effects of physical competence's reduction because of fatigue, rather than MS's functional disability (16,17) on their employability (10,13,14,15,16,17). Even though some of them decided to leave (15) their job before getting fired, this has a profound impact on their self-esteem and changes the balances within the family (15). Men seemed to be affected more from that because they become reliant on their wife's income (15) and their self-identity gets challenged (15). For instance, a male participant had decided to stop working, but the stress of this event triggered him a relapse, immediately afterwards: "When I finished up, I come home... and something snapped here, and I ended up on the floor. I couldn't even walk" (15). Some participants replaced their demanding work with a part time job (17), while others chose a social activity for coping with work loss (10).<br><br>**VERBATIM STATEMENTS**<br>For example, participant #2 stated: 'I have coped with not being able to work by taking care of the kids in the neighbourhood. (10)<br>Another participant realised that exercise was no longer helping to achieve her goal of maintaining enough stamina to remain in fulltime employment. (13) | 10, 13, 14, 15, 16, 17 |

| | | | |
|---|---|---|---|
| | | Participants also described fatigue as affecting aspects of clients' lives including employment, relationships, personal expectations, and ability to follow health-related recommendations. (14)<br>Loss of employment had a profound impact on another man, who made a decision to leave his job before he was fired. He described how the stress of this event triggered a relapse of his MS on arriving home afterward: "When I finished up, I come home . . . and something snapped here, and I ended up on the floor. I couldn't even walk." (15)<br>For example, one man lost his job and became more reliant on his wife for household income. (15)<br>Twin losses of driver's license and employment challenged men's self-identities within the family. (15)<br>The loss of activity also had a direct impact on employment. (16)<br>" Another informant, a talented, professional woman, talked about the serious decisions and drastic lifestyle changes she had to make in response to her severe MS-related fatigue. Careful probing revealed that fatigue, rather than functional disability, had required her to give-up her position as director of a non-profit organization, resign from a part-time college teaching position, and restrict her clinical practice to 16 hours per week. (17) | |
| | Depression | This sub-theme identifies the relationship between fatigue and depression. Participants reported that they felt depressed when disengaged from meaningful activities or felt anxious for the future. A total of six studies supported this sub-theme (8,9,12,13,14,15). Across the studies participants reported that fatigue impairment provoked depression feelings (12,13,14) when realised their inability to achieve their goals (15), compared themselves with healthy people (9), felt fear for the future (13), or when disengaged from physical activities (8). There is a two-way correlation between fatigue and depression. In many cases depression presented with other fatigue consequences as cognitive problems, lack of balance, energy, or muscle weakness (13,14). There is a two-way correlation between fatigue and depression. Participants explained that depression increased fatigue symptoms (12,13) which cause further fatigue. For instance, a participant stated that: "a feeling of depression, which involved a fear for the future, and led to not being able to think about normal things: 'everything goes to pot' including deterioration in balance, walking and increased heaviness in the legs" (13). However, the frustration related with inability to achieve goals in some cases may be the onset of valuable adaptations (15).<br><br>**VERBATIM STATEMENTS**<br>For example, if someone had engaged in a lot of physical activity prior to MS, it did not necessarily follow that they would continue engaging in physical activity post diagnosis; as is clear from the experiences of one participant described above whose depression resulted in disengagement post diagnosis (8)<br>We're all the same, there is nobody any different, which was a bonus, because sometimes it's quite, when you see super fit people, it's a wee bit. . . (A4); Depressing, maybe? (9)<br>For example Katherine explains: 'Well, it tends to . . . my frame of mind as well, you know if I can sink into quite deep depression as well and when I feel really low I tend to not have a lot of energy, and then other days I can be feeling really good and yeah I feel sort of happy and full of energy' (Katherine, I1, P1). (12)<br>Participants described different states of fatigue including muscle weakness, a mental fogginess, feelings of depression and an all-consuming fatigue. Furthermore, many participants had experienced more than one state of fatigue during the course of their MS and, on occasion, one state of fatigue could trigger another. For example, Angela described a | 8,9,12,13,14,15 |

| | | | |
|---|---|---|---|
| | | feeling of depression, which involved a fear for the future, and led to not being able to think about normal things: 'everything goes to pot' including deterioration in balance, walking and increased heaviness in the legs. 13<br>MS-related fatigue was perceived as having very little scientific explanation and often seemed inextricably linked to other MS-related impairments such as depression, poor balance, and cognitive difficulties. 14<br>Frustration, if ongoing, can lead to depression in some people, and Carver and Scheier (2000) point out that "the depression response is most likely to occur when the frustrated goal is deeply connected to the core of the self. Such a goal is not easily abandoned or even scaled back" (p. 1721). Carver and Scheier also suggest that lapsing into depression might be a reflection of slower recalibration of goals. 15 | |
| | Type of exercise | Definition of theme: This sub-theme identifies the perceived difficulties or facilitation from particular type of exercises corresponding with their symptoms.<br><br>Participants with physical deterioration avoid vigorous exercise such as aqua aerobics, chair-based yoga [1] or classic yoga [2], running [2], normal road bike [2], and walking outdoors [13] of the fear of fall. Some of them choose stationary cycle [13] and treadmill [13] which gave them a sense of safety because "there's no paving stone to trip over" and also may hold onto bike/treadmill if they need [13]. Across the studies participants avoided [2, 9, 10] or chose [3, 13, 15] to participate in the same activities for just the opposite reasons. For example, participants with poor balance reported feeling unsupported and anxious to exercise in swimming pools [2] while another stated that when exercised in water gained a sense of normality [13]. Some avoided swimming pools because are warm [10] while others chose swimming for staying cool [15]. Moreover, swimming increased the energy levels in PwMS [8], exacerbation of MSRF with the swimming reported in 2/19 studies [3,8]. Participants mentioned the significance of monitoring their fatigue levels to be able to continue and enjoy their activity [3]. Endurance exercise increases body temperature, so often was avoided by individuals as this increases MSRF [18].<br><br>**VERBATIM STATEMENTS**<br>Participants with functional decline or fear of falls reported having difficulty with finding a safe way of doing vigorous exercise: "I was doing aqua aerobics and chair-based yoga as well, but I had to stop because I deteriorated" (P3.1, 57 y, sev) (1)<br>Well, on the few times I have tried to run, I can do about half a mile, good half mile and then the legs do start to become weak after running for half a mile and my feet start to trail a little, I have a job to, I can't pick them up, I'm in danger of tripping, that's not the case when I'm walking, I could walk for oh near two miles and then the legs would just become tired, they wouldn't start tripping me up as they do when I run. (2)<br>Lesley spoke of how balance affected her exercise experience, but experienced physical problems which resulted in a negative cognitive experience: *I have problems with balance which is one of the reasons why I found swimming difficult because I felt unsteady,* unsupported in the water and anxious that I was going to do a nosedive. When I started trying to swim I went round in circles because the right arm is weaker but I learned to overcome this. (2)<br>Nathan described how his lack of balance dictated the type of exercise he does: I use a treadmill. I do a 15 min walk on the treadmill, the reason I use a treadmill is because you've got the arms at the side that you can hold onto if you need to and the arms at the front, I try not to hold them but if my balance is going I can get hold of them. (2) | 1,2,3,8,9,10,13,15<br>,18 |

| | | | |
|---|---|---|---|
| | | The treadmill's not got any steps to go up, it's all flat and it's level and there's no paving stone to trip over or anything like that. I use the exercise bike as well, if I used the normal road bike I'd worry about falling off when going round the corners or having to stop or something when people walk out in front of you and on the exercise bike you're just sat there. (2)<br>Claire described how problems with co-ordination affected her exercise experience and loss to exercise identity (e.g. not being a good swimmer anymore): I used to be a good swimmer when I first started, my co-ordination one of the worst affected things 'cause I literally couldn't co-ordinate my arms and my legs together. I don't know whether it was because I was frightened and unstable indirectly but I couldn't even relax and float. (2)<br>Lesley also described how her poor balance affected her during exercise: I went back to doing some yoga than, I learnt about this lady she did it from her own home, so I went there. I had to give that up, my balance was so poor there wasn't the space, I could have perhaps carried on a bit more if I was able to wobble about a bit, but I wasn't so I gave up going to yoga. (2)<br>Aquatic therapy: "It makes you feel good, you miss it if you don't go. . . . Your body lets you know, like especially if you haven't been at it for a while. ….. Participants discussed the importance of monitoring their fatigue levels while in the pool so that they could continue to enjoy the program. (3)<br>'With the swimming, I did have to kind of persevere a little bit because the first few weeks I felt absolutely knackered. So, most of the rest of the day you know . . . ummm . . . But I think about six months in I realised that I was overall feeling a lot more energetic'. (Cheryl, Age 41, Relapsing remitting MS) (8)<br>I did do a programme of exercise, a lot of swimming and sit-ups to build the muscles up. But MS kicked in and I wasn't able to do any of that anymore, well I just kind of stopped doing anything. (9)<br>Some reported that the swimming pool was too warm or that the facility was not easily accessible, and that getting there increased their fatigue. (10)<br>Angela depicted her experience from aqua jogging: "I think in the water you feel like a human being again. You feel like you're normal, whereas on land you don't feel normal. I think it's the feeling as you're on an equal footing with everyone "(13)<br>Louise, who had previously experienced a minor relapse whilst walking for exercise, described why she then chose to use the stationary cycle: 'I wouldn't want anything like that to happen out of something I was doing to try and make things better'.(13)<br>One man described how swimming several times a week allowed him to stay cool during exercise. (15)<br>Participants often cited raised body temperature as a barrier to exercising for endurance, as this added to fatigue. (18) | |
| | Stress<br><br>Colour scheme:<br>Red: stress and fatigue<br>Green: increased stress inhibits exercise | This sub-theme identifies the relationship between stress, fatigue and exercise. Participants related stressful events with fatigue and most of them stated that when was increased their stress was also increased and fatigue perception. In addition, the less active participants believe that physical activity increase their stress while others chose to exercise for stress management<br><br>Across the studies participants described the distress of fatigue experience (14,17) and many of them attributed fatigue on psychological stress related to family, work or emotional problems (10,13,15,17). For instance, a participant stated: "I think inner personal stress creates fatigue" (17). In addition, participants assumed that a stressful event caused a relapse | 10,11,13,14,15,17 |

| | | | |
|---|---|---|---|
| | Blue: exercise reduce stress<br>Yellow: stress cause relapse | (14,15). A participant explained how made the decision to stop working before was fired and when resigned and returned home, experienced a relapse (15). Control over fatigue might cause an increase in their stress because of a potential exacerbation of their symptoms (13). Participants with low level of physical capacity mainly those who experienced stressful factors in their life reported difficulties to participate in physical activities and some adopted negative coping styles (10). In contrast, other participants were engaged to exercise to reduce their stress level (10,11,13).<br><br>**VERBATIM STATEMENTS**<br>But because the stress level was high, the responsibility was more. Not just because of the heat but because of everything, the stress, just the whole, all of it built into one summer. I was like ehhh; you know the summer from Hades made it difficult to exercise. (10)<br>All three inactive participants reported considerable stress in their life. For example, participant #11 felt guilty about not exercising, but also felt that coping with her brother having cancer, changing jobs, and recovering from an exacerbation made exercise too difficult. Participant #12 frequently had to take care of her grandchild, which she believed increased her stress and fatigue. (10) In addition to these stressful events, participant #11, #12, and #13 displayed negative dispositional coping styles, such as being in denial about MS, avoiding MS-related functions, and expressing helplessness over the disease. (10) On the other hand, active participants displayed positive and engaging coping styles, which enabled them to participate in social roles that they found meaningful. (10)<br>I just exercise to keep mentally well…it's for me and to keep me sane and um reduce my stress level…I need some downtime and that's the way I get it. I'm just getting some physio and some exercises you know to try and gain my strength….I feel good that I've done that for myself like you know it, it's psychologically good for me I think, I feel I'm doing whatever I can do to improve and keep myself on track and that's important I think to know that you're doing all you can do to keep your quality of life intact. (10)<br>One can see that physical activity is used for more than just the physical benefits, but also to improve their psychological health by providing an outlet for stress management and to improve self-esteem and self-efficacy. (11)<br>If exercise choices reinforced self-integrity then perceived control over the beast was increased; however, increased stress, often in the form of new or exacerbated symptoms, could reduce perceived control over the beast. (13)<br>Control over the beast could also be compromised by an increase in stress, in the form of disease exacerbations or ordinary problems in everyday life. Each of these themes will be discussed in more detail in the following section. (13)<br>Several participants were also motivated to exercise by their experience of symptomatic and psychological relief following exercise, including improved fatigue, physical sensation, mood, relaxation and pain. (13)<br>Ensuring both physical and psychological safety was important to all participants in this study. In addition, several participants believed that they could avoid certain triggers of relapse such as over exertion or stress.<br>This process required strategies that ensured the client was aware that his or her concerns were heard and included the use of imagery to try to understand and/or identify with the fatigue experience; for example, one participant compared MS-related fatigue to the fatigue experienced during menstruation and another to posttraumatic stress syndrome. (14) | |

| | | | |
|---|---|---|---|
| | | Loss of employment had a profound impact on another man, who made a decision to leave his job before he was fired. He described how the stress of this event triggered a relapse of his MS on arriving home afterward: “When I finished up, I come home . . . and something snapped here, and I ended up on the floor. I couldn’t even walk.” (15)<br>Increased fatigue was also attributed to psychological stress related to family, work, socioeconomic, and other emotionally laden problems. One participant noted, "Stress can really make me feel tired . . . on hard emotional tasks, at the end of them, I really feel drained. (17)<br>Another said, "You know, I just found that when you worry, you just shut down." A third said, "I think inner personal stress creates fatigue." (17)<br>Diversional activities were often used to distract attention from the distress of the fatigue experience and included listening to music, light reading, and talking on the telephone with friends. (17) | |
| | Increased duration of activities<br><br>Colour scheme:<br>Red: takes longer<br>Yellow: consequences<br>Green: worsening<br>Blue: emotional effects | Definition of theme: This sub-theme identifies an increase on the duration of physical activities, as a consequence of fatigue and MS impairments.<br><br>Across the studies participants described their difficulty to perform physical activities as rapidly as they were able to do them before MS diagnosis (2,7,15,16). This impairment is related to energy loss (15), it seems to get worse on early morning activities (7,16) and influences the individuals both physically and emotionally (2,7,15,16). Several participants felt embarrassed with the elongation of simple daily activities, as for example washing and dressing themselves (7,16). However, some of them expressed their satisfaction and a sense of accomplishment because they were still able to work or perform their daily tasks (7,15). A participant stated that: “I can’t go as quickly as I did in 2005; it takes me 3 times as long to do a job” (15). MS individuals explained how this impairment inhibits them exercising publicly (2). Even though they were still able to perform specific activities, they slowed down their peers who were behind them (2) and consequently some of them choose to exercise on their own (2).<br><br>**VERBATIM STATEMENTS**<br>It does limit me. I mean I can do it, I can walk but it takes me such a long time and it takes so much out of me, cause if I do that, something else has to go exercise wise, if I am at home on my own. (2)<br>I’d like to be able to play golf, still play golf. Which I played a few times last year and I had a buggy to get around the course and became increasingly difficult and was very, very slow, so holding everybody behind. (2)<br>Early morning is not good for me because, frankly, getting dressed is a major accomplishment really. I sort of think ‘Ah, I’ve done it!’ but it takes so long because early morning things are a little bit difficult for me. (7)<br>Loss of energy was described in the context of slowing down as it always took longer to complete a task. One man described his experiences herding cattle: “I can’t go as quickly as I did in 2005; it takes me 3 times as long to do a job.” (15)<br>… when you were fit you used to sort of get washed and dressed in about half an hour; in your greatness, but now you’ve got something that’s holding you back all the time, you know it’s going to take at least three quarters of an hour to an hour to get washed and dressed and up and out of bed …which is ridiculous when you look back at it. (16) | 2, 7, 15, 16 |
| | Social isolation | Definition of theme: This sub-theme identifies how the perceived fatigue inhibits form participation on exercise and social activities. | 2,7,10,17 |

| | | | |
|---|---|---|---|
| | | Across the studies participants believe that fatigue affected their performance in sport and considered not socially acceptable their behaviour, so excluded themselves from exercise (2,17). Other experienced restrictions on physical capacity which limited them to engaged in social activities (2,7,10,17).<br><br>**VERBATIM STATEMENTS**<br>…was concerned about slowing other golfers down, meaning that his behaviour is no longer socially acceptable given that he 'holds' 'normal or able-bodied' people back from enjoying their sport. The social implications of having MS are that the person eventually will exclude themselves from of sport/exercise/physical activity. (2)<br>The loss to social connectedness through sport was one of the consequences of MS, coupled with loss of exercise enjoyments like 'runner's high' and not being outdoors. (2)<br>…. There are two friends of ours that we're very close to, but I hadn't been to their places in a year because both of them have a lot of stairs in the houses, but now I'm back to visiting them. … It also illustrates the social implications of MS, and the extent to which faltering physical function can limit social opportunities. (7)<br>All of the spouses were particularly attuned to the notion of isolation, and the tendency for their partners to withdraw from social activities. Betty (spouse) explained: I realized that he was staying at home, he wasn't doing anything, he was isolating himself and I think that's both emotional and physical. He didn't feel well with the fatigue, it hurt to move, he didn't want to do anything, he didn't want to go anywhere. (7)<br>For example, participant #13 did not like going to the wellness group sessions because of her fatigue… (10)<br>Many participants talked about working fewer hours, no longer enjoying favourite social activities, not applying for promotions, not entertaining according to spousal expectations, difficulty getting housework done, and omitting activities or leaving activities early to ensure the necessary amount of sleep to preserve existing energy. (17) | |
| | Effects on Spousal relationship<br><br>Colour scheme:<br>Red: spousal relationship<br>Yellow: emotional response<br>Green: spousal increased commitments | Definition of theme: This sub-theme identifies how fatigue influences MS individuals' relationship with their partners<br><br>Across the studies participants described fatigue as affecting their relationship (14), increase their tendency for isolation, their inability to accompany spouse in social activities (7) and their inadequacy to meet spousal expectations (17). MS individuals' spouse described how were both affected from the physical and mental effects of fatigue (7), had guilty feelings when enjoyed activities solely (7), and felt overloaded with housework, family commitments and partner support (7,17). Conversely, MS individuals perceived dependency for household spouse income and support, so they avoided asking things even though they were important for them (15). For instance, a participant: "As a result of this perceived dependency, he was unwilling to ask his wife to drive him to exercise classes in the city" (15).<br><br>**VERBATIM STATEMENTS**<br>John (spouse) acknowledged how close he and his wife were to a complete breakdown of their relationship, due to her deteriorating condition and the mental toll it was taking on both of them. We were both to the point where we were almost ready to divorce or separate because of her disease taking her down. I had to do everything, all the housework, and laundry and sometimes helping her get dressed and for a while, after she broke her ankle, we were really, really in a bad place for a while. (7) | 7, 14, 15, 17 |

| | | | |
|---|---|---|---|
| | | I felt guilty going and leaving her, getting out and doing something and not being able to take her. (7)<br>All of the spouses were particularly attuned to the notion of isolation, and the tendency for their partners to withdraw from social activities. (7)<br>Participants also described fatigue as affecting aspects of clients' lives including employment, relationships, personal expectations, and ability to follow health-related recommendations. (14)<br>For example, one man lost his job and became more reliant on his wife for household income. As a result of this perceived dependency, he was unwilling to ask his wife to drive him to exercise classes in the city. (15)<br>Many participants talked about working fewer hours, no longer enjoying favourite social activities, not applying for promotions, not entertaining according to spousal expectations, difficulty getting housework done,… (17) | |
| Facilitators to exercise | Feeling generated within exercise<br><br>Colour scheme:<br>Red: task<br>Yellow: physical improvement<br>Blue: feelings generated<br>Pupil: negative effect of inactivity | Definition of theme: This sub-theme identifies the perceived feelings generated by exercise. Participants reported that the increase of their physical capacity resulted in a sense of control over MS, improved their mood and normality and made them able to be more engaged in social activities.<br><br>Across the studies participants associated the engagement in physical activities with a variety of valuable positive feelings (1,2,3,4,5,6,7,11,12,15,16). To start with, two participants stated: "Exercise feels good and this provides motivation: I enjoy feeling fitter" (P6.3, 39 y, mild), "The more exercise you do, the more you want to do" (P5.3, 63, mod) (1). Participants highlighted the development of a sense of control (1,2,12,15) which proved to themselves that they could do as much as they did before MS diagnosis (2,6). A participant explained the importance of this sense of normality (6,16): "What's been great for me, that I realise I am pretty normal ... and feel confident" (6), while others related this sense of well-being with improved mood and happiness (5,6,7). Many of them reported they gained a sense of accomplishment (1,5,7,12,15) and felt proud of themselves (1), even though the intensity of the activity was lower, or they had performed some adaptations in order to complete the task, and finally they experienced positive feelings (7,15). Another participant stated: "I just exercise to keep mentally well…it's for me and to keep me sane and um reduce my stress level" (11). The reduction of perceived sense of fatigue enabled them to feel stronger, with less floppy days (3,4,5) and more socially connected (7,16). A participant explained: "…my energy…I'm not as tired, not as heavy, I used to feel so heavy in myself and dead in myself, I feel I'm lighter and more flexible." (4)<br><br>**<u>VERBATIM STATEMENTS</u>**<br>Participants spoke at length about the physical feedback that exercise provides. Exercise feels good and this provides motivation: "I enjoy feeling fitter" (P6.3, 39 y, mild); " …Exercise is getting easier as [I] go. I think the more you do, the more is it the [endorphins]? …The more exercise you do, the more you want to do" (P5.3, 63, mod). (1)<br>… by creating a sense of control: "When you get back on track with the exercise you feel yourself walking better so you think oh good that's working…" (1)<br>Participants spoke of having a sense of accomplishment having completed challenging or endurance activities: "I feel so proud of myself after I've done a long bike ride." (P3.2, 57 y, mild). (1)<br>I feel by remaining physically active, I feel I am doing something to control the MS, whether I am or not, I don't know but I am keeping my muscles working and keeping my sort of legs toned and my arms toned. (2) | 1, 2, 3, 4, 5, 6, 7, 11, 12, 15, 16 |

| | | | |
|---|---|---|---|
| | | …as though you are doing something, like maintaining a life, or lifestyle you've got. Nathan is using exercise to try and gain a sense of control over MS, and he feels it is important to be able to prove to himself that he can do as much as he could before he had MS. (2)<br>So it's keeping your activity level up and your strength. . . . If you stay away for a long time you lose it totally… but if you keep it up you're in good spirits and you're in good health and you're stronger. (3)<br>Several participants described "feeling lighter" to represent a feeling of reduced fatigue: A2 "looking back I just be thinking about the amount of energy that it kind of gave me"; B3 "my energy . . . I'm not as tired, not as heavy, I used to feel so heavy in myself and dead in myself, I feel I'm lighter and more flexible." Participants also reported improvements in hand or leg strength and flexibility which also led to an increased ability. B3 said "well its good for our well-being and we feel better that we're able to do things…(4)<br>"I think I felt generally stronger" (T2), and "I know for me, I felt I was a lot straighter, standing straighter" (FG1:2). Daily physical functioning was also improved…In addition, there were reduced feelings of fatigue: "I feel as though I'm less fatigued. I don't have as many floppy days…. The acute health benefits of participating in the exercise sessions were also reported, including mood enhancement and a sense of well-being and achievement. (5)<br>An improved mood or happiness was mentioned as a positive outcome by six participants… Also possibly related to the improved mood of the participants were feelings of improved confidence (reported by four participants) and normality (reported by three participants). Participant 3 said 'what's been great for me, that I realise I am pretty normal . . . and feel confident'. (6)<br>The improved confidence and feelings of normality resulted in feelings of being able to participate more in societal roles for three participants. (6)<br>Spouses reiterated the positive effect that exercise had not just on their partner's physical health, but also on their attitude. He comes home positive. He feels better about himself, he feels that he's accomplished something. He notices that he can do things better, just simple tasks that he would do every day that he feels more confident about. Even our interpersonal relationship, he's more positive, he's more engaged. He comes home happier. (7)<br>I felt a little bit freer. I'm starting to get a little bit of independence back….(7)<br>I just exercise to keep mentally well…it's for me and to keep me sane and um reduce my stress level…I need some downtime and that's the way I get it. I'm just getting some physio and so me exercises you know to try and gain my strength….I feel good that I've done that for myself like you know it, it's psychologically good for me I think, I feel I'm doing whatever I can do to improve and keep myself on track and that's important I think to know that you're doing all you can do to keep your quality of life intact. (11)<br>In participants with a well-developed sense of control over fatigue, working beyond 'the edge' was associated with positive feelings of achievement. (12)<br>Those men who were able to engage in highly valued activities—even if the intensity was less, or if they achieved them through a different_route—experienced positive feelings and a sense of control. (15)<br>While exercise and physical activity were noted as having many benefits, it is apparent that these moved beyond the physical and the psychological to also include social connectivity. (16)<br>…exercise and physical activity were used as a way of coping with MS. These activities when utilised enabled a sense of normalcy and added structure to their lives: classes do sort of, give a framework to my week. (16) | |

| | | | |
|---|---|---|---|
| | Belief that exercise increase energy levels<br><br>Colour scheme:<br>Red: exercise increase energy<br>Pupil: exercise negative impact<br>Green: inactivity negative impact<br>Yellow: other benefits of exercise | Definition of theme: This sub-theme identifies the benefits of exercise on perceived fatigue. Although many participants suffered from the short-term negative effect of fatigue, recognised the physical, mental and psychosocial benefits achieved through physical activity and remain motivated.<br><br>Across the studies participants reported that taking part in physical activity creates energy (1,8,11,13,17), and increases the control over fatigue (10,11,13,17). Participants reported that even though they sometimes initially experienced a rise of fatigue, they felt better when they continued exercising (8,13,15). In addition, some of the participants described how, when they felt tired, needed to exercise and then experienced an increase in their energy levels (1,8,11). For instance, a participant stated: "You know, like even when I was really ill and incredibly fatigued, I did know that even if I shuffled to the end of the block and back, I felt a bit better" (8.) Conversely, if participants remained inactive, they had experienced lower energy levels and physical deconditioning (8,17). A participant depicted the perceived results of inactivity: "…exercise actually improves the fatigue level. But if I just sit and do absolutely nothing, I am actually more tired than if I do a little bit of exercise" (17).<br>Others perceived benefits from exercise: made them feel better (8,13), improved long term fitness (8,12,13), increased strength and endurance (13), made the legs feeling less heavy (13), gave a sense of "healthy tiredness" (3,13), and lead to a greater participation in physical activity (8,12,13,14).<br><br>**<u>VERBATIM STATEMENTS</u>**<br>I know that it creates energy for me so if I'm tired, I know I need to exercise" (P3.2, 57 y, mild). (1)<br>A certain degree of fatigue was noted to be positive, with participants viewing it as an indicator of having exercised and "used their muscles,"…(3)<br>They could see that taking part in physical activity would help increase their energy levels, but they just could not muster the energy to get going in the first place. (8)<br>That is, physical activity results in increased energy levels, subsequently **leading to a greater likelihood of participation in physical activity**. Conversely, inactivity results in lowered energy levels and physical deconditioning, leading to further inactivity. (8)<br>'Once you know that it makes you feel better, that **motivates you to keep going**. You know, like even when I was really ill and incredibly fatigued, I did know that even if I shuffled to the end of the block and back I felt a bit better'. (8)<br>However, those that persevered through the initial, short-term negative impact of physical activity reportedly experienced **long-term benefits.** (8)<br>Participant #5 had a goal of exercising three times a week because she felt it would help reduce fatigue. (10)<br>I find that I feel I have more energy because that's also one of the symptoms that affects me is the fatigue um and I find that I can get very drained very easily and I just find with doing any physical activity that that so to speak, pumps me up. (11)<br>….participants with a strong sense of control who sometimes chose to go beyond 'the edge' as they felt this was necessary in order to improve **long term fitness.** (12) | 1, 3, 8, 10, 11, 12, 13, 14, 15, 17 |

| | | | |
|---|---|---|---|
| | | Physical improvements included a perceived increase in energy levels, greater strength and endurance, less heaviness in the legs, a 'healthy tiredness', a 'healthy pain' and improved sleep. Both 'healthy pain' and 'healthy tiredness' were perceived as normal sensations associated with exercise, in contrast to fatigue and the 'MS pain'. (13)<br>Several participants were also **motivated to exercise** by their experience of symptomatic and psychological relief following exercise, including improved fatigue, physical sensation, mood, relaxation and pain. (13)<br>All participants in this study, including those who sometimes experienced increased fatigue associated with exercise, believed that control over the beast was possible through the physical, mental and psychosocial benefits achieved through exercise. (13)<br>Some participants, whilst recognising their limits, chose to go beyond them despite suffering temporary physical deterioration such as increased fatigue. By going beyond their limits, these participants experienced either long-term fitness gain or achieved goals. (13)<br>….exercise participation could help to strengthen this positive attitude and **increase motivation.** (14)<br>…..he would often compromise on his exercise when he felt fatigued and yet occasionally would think "ah, no-bugger [an expletive] this" and continue with usual activities regardless of his exhaustion. (15)<br>Another individual noted that, "exercise actually improves the fatigue level. But if I just sit and do absolutely nothing, I am actually more tired than if I do a little bit of exercise." (17) | |
| | Group exercise<br><br>Colour scheme:<br>Red: group exercise scheme<br>Blue: benefits<br>Pupil: social benefits<br>Green: future benefits | Definition of theme: This sub-theme identifies the perceived benefits from group exercise including: motivation, learning, positive emotional response and socialisation.<br><br>Across the studies participants highlighted the value of group exercise to develop a sense of motivation and support (4,7,15,19). They reported that it was easier to exercise in a group than at home (4,7,19) but expressed their belief that the team contributed to continuing exercise at home (4). Participants emphasised the importance of exercising with individuals who had similar difficulties (6,7,9,10,16,19). They gained new learning and experiences by sharing their mutual problems, as well as through the interaction amongst the group (7,19). Exercise peers and group leaders provided them encouragement and inspiration to try harder (6,7,9) Participants reported many positive emotional responses as they felt: safe (6), confident (6), encouraged (6,7), empowered (9,19), their attitudes improved (7), they stopped feeling sorry for themselves (9), they were "liberalised and normalised their daily life" (13) and accepted by others (13). However, the social aspect seemed to play a pivotal role on participants perceived benefits (5,6,7,10,19). For instance, a participant stated: "I really enjoyed the social aspect of meeting people and talking because you don't always get that when you have MS. You tend to be at home a lot on your own, so I enjoyed coming" (5). Participants mentioned more advantages as being part of a group: the relationship with other people (7,19), the facilitation of making new friendships (7,16), partnership (19), sharing stories with others (10) and the possibility to undertake leisure activities with them (16).<br><br>**VERBATIM STATEMENTS**<br>Participants talked frequently, extensively, and with emotion about how the group structure of the class served as a source of motivation and support. They talked about how it was much easier to exercise as a group rather than at home as the group motivated and supported each other. They also described the importance of the "team" and talked about how being a member of the team also contributed to motivating them to attend and to exercise at home. (4) | 1, 4, 5, 6, 7, 10, 13, 15, 16, 19 |

| | | | |
|---|---|---|---|
| | | In some cases, having a reason to leave the house was a valued benefit of taking part in the program: "I really enjoyed the social aspect of meeting people and talking because you don't always get that when you have MS. You tend to be at home a lot on your own so I enjoyed coming." (5)<br>…, five participants also valued the encouragement they received from the other members of the group as well as the leaders. (6)<br>A final social benefit of the programme was working in a group with other people with MS. Five participants found this a positive outcome. Participant 1 felt 'safe and confident working with others with MS on something that was new' (6)<br>The participants with MS, particularly those who were involved in6,7, the structured group-based exercise program, emphasized the social aspect and how that was beneficial above and beyond the exercise itself. Belonging is an important thing. You belong because you're doing the exercises and you're sharing it with everyone…. You're in a relationship with people, you can see them doing it and you want to work harder to do it yourself. (7)<br>The participants spoke favourably about group exercise and found this method provided an environment that supported the encouragement of one another, motivated, improved their attitudes towards disability and MS, and facilitated new friendships. (7)<br>I think because everyone is in the same boat, it stops you from feeling sorry for yourself, feeling oh I can't do this, you see everybody else getting on with it and it inspires you to try harder. (9)<br>… participant #11 liked going to the group sessions because she was able to share stories with other participants. (10)<br>Two participants described a sense of freedom associated with exercising in a community-based environment, as it both liberalised and normalised their daily life. (13)<br>A lovely class where everyone accepts that I have limitations so its great because if you can't do a certain exercise or you're only doing up to half the class, and just sit there in relaxation the whole time, people are very accepting. (13)<br>…in those men, who previously had preferred to exercise alone yet found additional benefits exercising in a group. (15)<br>Firstly, by the social interaction usually brought about through exercise classes that provided space to engage in friendly banter and peer support. Secondly, through social connectivity, that is, connecting with others through the capacity to undertake leisure activities: well, you know, you're getting out and about you're seeing people and meeting people, you're keeping up some social contacts at the same time. (16)<br>… participants described an experience of empowerment and partnership: ' . . . it wasn't so much a group of people being talked to . . it was very much a group of people talking together . . ' (Fiona). (19)<br>Participants described working in partnership with the programme facilitators: 'The person that's giving the course is part of the course, and is one of you nearly, and is there to help you, not to sort of drum a thing into your head'. (19)<br>The social experience of being part of a group with a shared experience of fatigue was discussed by all participants. Participants described the group experience as 'absolutely essential' (Peter), 'a great benefit' (Emie) and 'very useful' (Norma). The sharing of experiences and interaction amongst the group facilitated new learning and 'the sharing of ideas' (Kevin). The sharing of expertise was described as beneficial because 'you were learning from others as well as [from] the people who were giving the course; you learned as much nearly from the people around you' (Kate). (19)<br>Participants described the support obtained from meeting others with the same condition and experience: 'It's fantastic to see other people that know exactly what you are talking about and you just click with them because you just know | |

| | | | |
|---|---|---|---|
| | | what each other is feeling' (Ruth). ' . . . we were all on the same boat, just that support was great . . . that wasn't [from] the programme, that was a side effect of the group' (Fiona). (19) | |
| | Appropriate level &/or type of exercise<br><br>Colour scheme:<br>Red: choose exercise<br>Blue: due to Pupil: not exercising to "the edge" consequences<br>Yellow: outcomes<br>Green: reason for choosing exercises | Definition of theme: This sub-theme identifies the significance of making necessary modifications on the type and intensity of their exercises to be able to maintain a suitable level of physical activity.<br><br>Across the studies participants described their need to scale back the intensity, duration and frequency of exercise (1,9,10,11,12,15), and also to choose between a variety of options the most suitable exercise, which minimised their physical symptoms (2,9,10,13,15). For instance, Angela depicted her experience from aqua jogging: "I think in the water you feel like a human being again. You feel like you're normal, whereas on land you don't feel normal. I think it's the feeling as you're on an equal footing with everyone "(13); another participant weighted a pamphlet delivery cart with bricks in order to gain greater physical support whilst walking" (13). Equally important was the choice of exercise settings mostly for the participants who experienced high levels of fatigue and lack of balance (2,10,11,13,15). A broadly used choice was exercising home with gym equipment (10,13,15), where they experienced higher level of convenience, privacy and security with lower energy expenditure (13,15) and that enabled them to disperse exercise over the day or to stop when they felt tired (10).<br>In addition, participants had strong control over their limits by listening to their bodies, wanted to further challenge themselves and needed to progress on different types or levels of exercise because they felt boredom and frustration when they were unavailable to push themselves to their real limits (9,12). Overall, all participants reported their willing to design their own programme and choose the type and level of exercise in a tailor-made physical activity, suitable for their temporary physical capacity (1,9,10,11,12,13,15,19), thus creating a balance between the perceived drawbacks and benefits of exercise (13).<br><br>**VERBATIM STATEMENTS**<br>Participants prioritized and scheduled exercise, exercised in small increments, focused on exercise they enjoyed doing, employed self-monitoring techniques using apps and devices, made a commitment to exercise (such as buying a dog that had to be walked), and where necessary, made modifications to their regimens in order to maintain some level of exercise. For example, participants related scaling exercise to cope with injury and recovery (chair yoga/yoga, recumbent cycling/cycling). (1)<br>Most participants had incorporated or increased their levels of incidental exercise (incidental walking, dog walking/training, gardening, playing with children) and/or gentle exercise (walking, yoga) since the intervention. (1)<br>"I suppose I've tried different forms of exercise… At the time I was doing swimming; I've done yoga as well. Yoga I really enjoyed but I'm not doing much of it at the moment… So I have experimented a bit with exercise to find the right thing." (P6.3, 39 y, mild). (1)<br>I use a treadmill. I do a 15 min walk on the treadmill, the reason I use a treadmill is because you've got the arms at the side that you can hold onto if you need to and the arms at the front, I try not to hold them but if my balance is going I can get hold of them. (2) | 1,2,9, 10, 11, 12, 13, 14, 15, 19 |

| | | | |
|---|---|---|---|
| | | Participants were positive about the range of exercises included in the class and the choice of aerobic, resistance and balance options. They liked the varied levels of difficulty available for each exercise. I was really impressed with the fact that there were [exercise] options for different levels of difficulty…. (9)<br>Some participants felt that they were now ready to progress to different types of exercises to alleviate both boredom and challenge themselves further. I'd quite like a Pilates class for people with MS; I think that would be quite good because it is concentrating on your core muscles. (9)<br>On days when I was tired, I reduced the number of reps. I sometimes space exercise over the day'. (10)<br>Active participants typically described moderate to vigorous activity as PA. They believed that they were more physically active than their peers, and that they needed to engage in PA 3 to 7 days a week to stay healthy. (10)<br>Participant #3 observed that if she did not stretch everyday she would become stiff and would have difficulty walking. (10)<br>It was evident that there was a fine line between beneficial amounts of activity and overexertion, and it was up to them to prioritize the type and intensity of activity participation. (11)<br>Participants with strong control listened to their body and chose the level of intensity at which to exercise;……'Reaching the edge', a perceived safe duration, level and type of exercise for each individual. (12)<br>Staying well away from 'the edge' or 'playing it safe' led to feelings of boredom, frustration and consequently increased negative feelings associated with an unhealthy tiredness. (12)<br>And I was able to control it more I was able to push myself to what my limit was; not to what they thought my limit was' (Mitch. I3, P1). (12)<br>Feeling normal was an important experience for some participants and often determined activity choice. Angela describes her aqua jogging experience: I think in the water you feel like a human being again. You feel like 'you're normal, whereas on land you 'don't feel normal. I think 'it's the feeling as 'you're on an equal footing with everyone. (13)<br>…for whom exercise choice was self-defining recognised a state where they felt happy with the trade-offs between perceived benefits and drawbacks of exercise. (13)<br>Diane enjoyed walking; however, due to reduced balance, she had to concentrate harder on lifting her feet and fixing her gaze. This process demanded high levels of energy, thus Diane was concerned that she would tire before making it home. (13)<br>Other participants chose to exercise at home using home gym equipment for reasons such as increased security, privacy, convenience and being too self-conscious to exercise outside the home. (13)<br>… one participant felt more physically supported in the water whilst aqua jogging; another participant weighted a pamphlet delivery cart with bricks in order to gain greater physical support whilst walking. (13)<br>Sam enjoyed yoga because 'It's not about everyone else it's about you'. (13)<br>Diane, who describes herself and her family as having a love of the outdoors, describes walking by the beach: 'Just the fishing . . . the water, the fresh air, beach combing . . . my mind was a totally different place um than walking down the street . . . '(13)<br>For example, one participant who taught Pilates to a group of people with MS described the influences of improving posture on mood: So I think of it that perhaps with Pilates, the exercise can perhaps affect the way they are feeling by | |

| | | | |
|---|---|---|---|
| | | increasing you know shoulders up, and head up, perhaps you can reverse it the other way and make them psychologically feel a little bit better with their bodies being held better. (14)<br>This type of scaling back, or reduction in the intensity (rate) and duration of exercise, was a common response to reducing fatigue and this man changed his response because he was unable to achieve his previous intensity of martial arts skills. (15)<br>Adjustment of exercise intensity was a strategy frequently used by the men. (15)<br>Choice of exercise setting enabled the men to self-pace activities, which seemed more important for those whose fatigue resulted in weakness and balance difficulties. (15)<br>Several men chose to walk with their dog because it helped them stick to exercise routines. One participant described needing "dog power" when he was losing motivation. A dog helped maintain engagement with walking, and several men implied they would not walk for exercise without a dog. In a prior study, women had described walking either alone or in groups with other human companions (Smith et al., 2011). Dog walking could be a useful intervention to explore, for example, the usefulness of dog-walking groups for men with MS. (15)<br>However, a small group of men in our study from one geographical region found strength and shared meaning at a yoga class hosted by the Multiple Sclerosis Society. (15)<br>When this man felt he was nearing his limit, he slowed his cycling speed or got off his bike and walked until he felt better. This strategy allowed him to achieve his "be" goal of continuing to be a cyclist. (15)<br>Participants identified lifestyle changes as the key modality to managing their fatigue: 'I've actually signed on for yoga' (Fiona). (19)<br>All participants described how the non-directive approach of the programme acknowledged their expertise and allowed participants' decision-making power, with information provided to support decision making…. 'On the very first day, they threw it back at us to try and design the program and I think that was very beneficial because it's only us that have the MS, so it's us that know what we need'. (19) | |
| | Adaptability and positive thinking<br><br>Colour scheme:<br>Red: target/ adaptations<br>Yellow: positive think<br>Blue: outcomes<br>Green: exercise related adaptations<br>Pupil: Copping strategies | Definition of theme: This sub-theme identifies the perceived improvement, including a development of control over fatigue and positive feelings, by planning ahead for pre-empting situations and making adaptations on meaningful physical activities upon the level of fatigue.<br><br>Across the studies participants reported many different strategies which empowered them to reduce fatigue experience and so to remain active (5,9,10,11,12,15,17,18), contribute a sense of optimism (9,10,11,12,15,16) and participate in social roles (10,16). Participants reported their attempt to "work smart" in order to maximise performance and minimise the energy cost (17). This tactic included strategies as "sit to do an activity rather than stand" (18), "try to make activities simpler" (18) or "omit an important activity to conserve energy for a more valued activity later in the day" (17). One of the participants highlighted the "need to come up with their own solution from the inside. Then they own it; they then believe in it and then it works for them" (17). However, some of these strategies imposed a readjustment on MS individuals' standards and expectations (18). These decisions may be related to the intensity, duration or type of exercise but also to the perceived value of the activity and allowed them to achieve a meaningful goal (5,12,15). A participant summarised in few words that: "Whereas I wanted to run she was still teaching me to walk, so from that point of view I learnt a huge amount and gained a huge amount, and that's how I am taking it forward" (5). | 5, 9, 10, 11, 12, 15, 16, 17, 18 |

| | | | |
|---|---|---|---|
| | | Participants that engaged in various coping strategies and approached positively over their experienced capabilities and independence, regardless of their level of disability, might have coped better with MS restrictions (11,12) and were empowered to participate in meaningful social roles (10,16). For instance, a participant stated: "I would put physical activity and socialising in, somehow overlapping in the same bracket. Because, I can't really do anything physically, but I can socialise, I can enjoy meeting people like you, chat and have a very fulfilled life, really" (16).<br><br>**<u>VERBATIM STATEMENTS</u>**<br>A small minority of participants were able to maintain regular exercise by becoming adept at pre-empting situations, planning ahead, and making adaptations to their lifestyle with a positive frame of mind. (5)<br>Others felt that with continued exercise they could improve more. We could all do better yet. (9)<br>… active participants displayed positive and engaging coping styles, which enabled them to participate in social roles that they found meaningful. (10)<br>…these women have not only accepted their condition but can be appreciative and grateful for what they do have, regardless of their stage or level of disability. The vast majority of women in this study mirrored this positive outlook and the impact it had had upon their lives while living with this condition, which helped them to better cope with their disease. (11)<br>…these women felt for their current level of health and for the capabilities and independence that still remained, they had an attitude of "it could be worse". This attitude seemed to contribute to their sense of optimism. (11)<br>From these findings it would appear that perceived physical improvements and positive feelings may reduce the fatigue experience….(12)<br>Recalibrating "do" goals involved many strategies, decisions, and exercise choices. The ability to recalibrate appeared not only related to the intensity or type of exercise but also to the value the men placed on the activity. (15)<br>…participants described a developing sense of control over fatigue which involved a process of gradual adaptation. This was not without 'struggle' and highlighted emerging acceptance that certain activities would have to be replaced or modified depending upon the level of fatigue. (12)<br>We propose that in a condition like MS, with symptoms such as complex fatigue, the ability to adjust "be" and "do" goals is a necessary and ongoing skill that might help preserve aspects of self-identity and quality of life. (15)<br>Symptoms of MS, particularly complex fatigue, necessitate frequent and ongoing response shifts, or readjustments of "do" goals, to prevent discrepancies between intended goals and the reality of current ability. (15)<br>He described having only "three blocks of energy in a day" and could tell how much he could expend and how much he would have left over. When this man felt he was nearing his limit, he slowed his cycling speed or got off his bike and walked until he felt better. This strategy allowed him to achieve his "be" goal of continuing to be a cyclist. (15)<br>Participants described a shift in their perceptions by learning that they do not need to exercise to exhaustion to experience health benefits, and that they could progress slowly at their own pace: "Whereas I wanted to run she was still teaching me to walk, so from that point of view I learnt a huge amount and gained a huge amount, and that's how I am taking it forward. Not pushing it to the limits where I'm dropping, but building it up very slowly" and ". you think I can't possibly do that and it just seems a huge obstacle, but actually now knowing that you can do a few minutes or a minute even at times and it will count, so it is achievable and attainable isn't it" (5) | |

| | | | |
|---|---|---|---|
| | | I would put physical activity and socialising in, somehow overlapping in the same bracket. Because, I can't really do anything physically, but I can socialise, I can enjoy meeting people like you, chat and have a very fulfilled life, really. (16)<br>One participant emphasized the need for each individual to come up with his or her own solutions. She stressed: [They] need to come up with their own solution from the inside. Then they own it; they then believe in it and then it works for them. (17)<br>Working smart is making choices that enable the individual to maximize performance or accomplish valued activities and minimize energy expenditure. This often means omitting an "important" activity to conserve energy for a more valued activity later in the day. (17)<br>Sit to do an activity, rather than stand was the third strategy equally-commonly identified as being regularly used. Apart from activities that would commonly involve sitting, people might choose to sit for other activities such as setting the table or cutting vegetables. (18)<br>Try to make activities simpler was the strategy second most frequently placed in this category (54.8%). Most participants reported that it was a strategy that they ordinarily used in their life; therefore, they automatically used it to manage their fatigue. Others said that they had already made activities simple in their lives, and, consequently, did not need to use it as a strategy to manage their fatigue. A common way to make activities simpler was to skip steps.<br>Some people explained that, in order to make activities simpler, they might have to change the standards that they would accept and expect. (18) | |
| | Priority shifting<br><br>Colour scheme:<br>Red: prioritise exercise<br>Yellow: purpose<br>Green: energy management | Definition of theme: This sub-theme identifies the importance of prioritization physical activities to be able to remain active. A wellness philosophy and the belief that exercise is a tool helping them to achieve their goals, support participants' decision to exercise regularly.<br><br>Across the study several participants prioritised exercise, as a part of their daily routine (11,14) because they felt better (11) and believed that exercise on daily base empowered them to maintain their functional improvements (14). Some participants decided to buy a dog in order to make a commitment to walk every day (1,10). Other participants reported that they carefully scheduled the type and intensity of exercise (11) and integrated them into their daily commitments (8), in order to enjoy exercising without overexert themselves (8,11,15). However, a man stated that there is a dilemma: "I can work less if I walk for about an hour ... It's all about compromise" (15). On the contrary of this dilemma the most active participants believe: "…that exercise was a valuable tool for improvement and maintenance of long-term health and well-being" (13), provided them the perception that "control is possible" and enabled them to accomplish their higher goals (13,15).<br><br>**VERBATIM STATEMENTS**<br>Participants prioritized and scheduled exercise, exercised in small increments, focused on exercise they enjoyed doing, employed self-monitoring techniques using apps and devices, made a commitment to exercise (such as buying a dog that had to be walked), and where necessary, made modifications to their regimens in order to maintain some level of exercise. For example, participants related scaling exercise to cope with injury and recovery (chair yoga/yoga, recumbent cycling/cycling). (1) | 1, 8, 10, 11, 13, 14, 15, 19 |

| | | | |
|---|---|---|---|
| | | 'You do have to plan a little bit as you say because like I am at the gym and so I will have to allow enough energy to get home, to be able to drive home, get inside, get the girls into bed. You know, there has to be sort of enough energy before I crash in the corner'. (8)<br>Because of these self-monitoring cognitions, active participants had a high level of commitment to accomplishing PA goals. (10)<br>Active participants typically described moderate to vigorous activity as PA. They believed that they were more physically active than their peers, and that they needed to engage in PA 3 to 7 days a week to stay healthy. (10)<br>It was evident that there was a fine line between beneficial amounts of activity and overexertion, and it was up to them to prioritize the type and intensity of activity participation. (11)<br>I know that every morning I need to do it and that's what I do, I have a routine every morning that I do. I think it's only going to make me feel better and get through this a lot easier than if I were to sit around and "veg"… (11)<br>As noted previously, all participants were exercising regularly at the beginning of the study. They all reported a 'wellness philosophy', which supported exercise, had a 'related goal' significant to the management of their MS for which exercise was perceived as useful and had a perception that 'control is possible'. They said that these three beliefs/values were critical to their decision to pursue regular exercise and thought that individuals who did not hold these beliefs/values would be unlikely to consider exercising on a regular basis (13)<br>Several participants chose exercise as a tool to help them achieve or maintain goals. (13)<br>Wellness philosophy involved the belief that exercise was a valuable tool for improvement and maintenance of long-term health and well-being. (13)<br>Several participants felt that functional improvements could be made by incorporating exercise into everyday life because "at the end of the day activity is exercise" (Participant 3). (14)<br>*"Do" goals.* More concrete "do" goals were related to how the men in this study would manage exercise in response to fatigue and low energy levels and described how the higher "be" goals would be accomplished. (15)<br>Another man describes this dilemma: *"I can work less if I walk for about an hour . . . It's all about compromise."* This is an example of another response shift strategy, or reprioritization of activities. (15)<br>Occupational changes were identified, including new strategies for performing activities, for example, 'planning and prioritizing' (Kevin). 'It just made me have time for myself and not to feel selfish, but to realize that this is the reality. I need to do this or that in order to maintain my health' (Norma). (19) | |
| | Acceptance of MS & Determination<br><br>Colour scheme:<br>Red: determination to be active<br>Green: result no PA | Definition of theme: This sub-theme identifies the importance of acknowledging the presence of MS which enables MS individuals to make appropriate adaptations on their lifestyle to achieve a high quality of life. These persons were determined to remain physically active, even though they fight with fatigue related symptoms.<br><br>Across the studies participants described how their determination to exercise empowered them to hurdle over the fatigue related barriers in order to remain physically active (2,7,8,10,11,13,14,15). Participants were conscious about the consequences of not exercising (10). Although most of them were not able to maintain their pre-MS level of activities (2,11), they continued exercising on a regular basis. Most of the participants reported that they should push themselves (11), to overcome the initial sense of fatigue (7), to manage with symptoms and other commitments (8,11,15) and to disregard the perceived fatigue after PA (10) for maintaining a regular exercise programme. (7). For instance, a participant | 2, 7, 8, 10, 11, 13, 14, 15 |

| | | | |
|---|---|---|---|
| | Yellow: not as active<br>Blue: accept MS | stated: "Your body doesn't feel the same you know um when I feel numb, numb and tingly, like I can honestly say that I don't really think it stopped me um but it [is] a reminder" (11).<br>This determination was the healthy consequence of the acceptance of the presence of MS in their life (11,14). These participants have decided not to focus on the limitations caused by MS, but they made the appropriate adaptations to optimize their health and quality of life (14) and fought to accomplish the highest possible standard for their life (10,11).<br><br>**VERBATIM STATEMENTS**<br>So yeah, I'm a lot less physically active than I used to be but I still try and keep active with my restrictions. (2)<br>Alex observed: Exercise is always a challenge, to make the hurdle over the initial fatigue to get into it, get into a routine. (7)<br>Those people who realised this were able to manage these symptoms in a way that enabled them to take part in physical activity. (8)<br>Active participants also focused on the positive aspects of engaging in PA and the consequences of not engaging in PA. For example, participant #2 wrote that although his fatigue levels increased after PA, he liked the feeling associated with engaging in PA. (10)<br>'I know that my fatigue will always be a problem, but I know that I can fight through it to accomplish my goals'. (10)<br>'Yeah, it's totally motivation. I mean, I come up with a lot of reasons not to exercise like, "Oh the weather's bad. It's too hot or you know, I'm too busy", but I just kind of come up with those excuses'. (10)<br>…these women have not only accepted their condition but can be appreciative and grateful for what they do have, regardless of their stage or level of disability. (11)<br>…most of the women stated that they had made a conscious decision to be physically active on a regular basis, although their definition of what constituted physical activity may have changed somewhat. (11)<br>I'm determined that I'll do it… You have to keep pushing yourself. (11)<br>Your body doesn't feel the same you know um **when I feel numb, numb and tingly, like I can honestly say that I don't really think it stopped me um but it [is] a reminder.** (11)<br>They have a sense of determination to lead a life in which MS is not the central theme, and where they can live their lives to the highest possible standard. (11)<br>… some participants who had observed progressive loss of mobility in family members were determined to avoid losing their own mobility, and strongly believed in the popular health message 'move it or lose it'. (13)<br>… clients had reached a point of acceptance where they were willing to acknowledge the presence of MS and make positive changes to optimize their health and quality of life. (14)<br>A participant described one attribute as a "Scottish Capricorn male thing" and believed this "pig-headed [stubborn]" quality helped him continue with both work and exercise despite fatigue. (15)<br>The determination of both men worked positively toward achieving their goals and was consistent with…. In this case, determination was an equally useful personality trait to have when overcoming the barriers of fatigue. (15) | |
| | Appropriate professional guidance | Definition of theme: This sub-theme identifies the perceived needs of MS individuals regarding exercise support and the benefits from appropriate professional guidance. It includes some of the used strategies from health care professionals. | 1, 4, 5, 9, 13, 14, 16, 18 |

| | | | |
|---|---|---|---|
| | Colour scheme:<br>Red: kind of support<br>Green: what need<br>Blue: positive effects<br>Yellow: professional strategies | Across the studies participants highlighted the significance of a person-centred professional guidance from an exercise specialist trained for a MS population (1,4,5,9) or a neurological physiotherapist (18). They wished for a professional competent for monitoring them, offer them feedback and advice (13), being able to understand their personal capabilities and limits (2,4,13,18), and suggest appropriate exercises adapted to MS variability (16). Participants reported the perceived benefits from this formal guidance as they: felt safe and supported (9,13), had their confidence increased (4), remained engaged in regular exercise (4,13), enjoyed and benefited from activity (9), learned how to pace themselves (5), were helped to recognise their limits (4,13) and memorised helpful tips (13). For instance a participant described his positive experience: "You are scared because you immediately get the symptoms from the increased body temperature and everything anyway, my feet automatically have pins and needles all up my legs and that is murder and it is a sign that I will have to stop, and in actual fact what I have learnt is that it will fade, that is alright, it is your body just reacting and increasing temperature and is perfectly normal and carry on" (5).<br>In addition, health care providers mentioned some of the creative strategies they used, such as the "active listening and careful observation" relied on their professional experience and required "sophisticated listening skills that identified implicit as well as explicit cues regarding client priorities, goals, and problems" (14). Another useful assessment tool was "fatigue diaries" which made the invisible symptom of fatigue more visible (14).<br><br>**VERBATIM STATEMENTS**<br>Person-centred and specific instruction was seen as helpful: "She [pilates instructor] knows what I can do and what I'm capable of and when [my body] is tired one week she'll be able to tell me that as I walk in." (P5.3, 63 y, mod) (1)<br>"I mean you really need the professional guidance along the way to give you confidence and keep doing what you're doing and remind you of the benefit of it." They also talked about now knowing what to do and what they could do, that is the knowledge of their personal limits as well as what exercises were appropriate. (4)<br>Participants felt that the exercise specialists taught them how to recognize signs that they are doing too much and how to pace themselves: "You are scared because you immediately get the symptoms from the increased body temperature and everything anyway, my feet automatically have pins and needles all up my legs and that is murder and it is a sign that I will have to stop, and in actual fact what I have learnt is that it will fade, that is alright, it is your body just reacting and increasing temperature and is perfectly normal and carry on" (5)<br>Participants felt that having exercise instructors specifically trained for a MS population, as in this study, allowed them to feel safe and understood. This helped participants to enjoy and benefit from the class. (9)<br>Individuals who felt safe and supported found their own community-based exercise programme and engaged in it regularly. (13)<br>Qualities that were associated with positive support included offering helpful tips, affirming healthy choices, helping to find limits, being treated as an individual, regular monitoring and not giving up. (13)<br>Despite frustration, most participants expressed the desire for more consistent understanding, monitoring and advice regarding exercise activity from both health care and leisure sectors. (13)<br>HCP participants in this study reported that they used a variety of **creative strategies** to inform and shape their practice. These strategies were labelled "creative" because they relied on personal experiences; working experience; trial and error; and observing, listening to, and identifying with the client. (14) | |

| | | | |
|---|---|---|---|
| | | An additional strategy used by participants in this study was a process of **active listening and careful observation**. This process appeared to involve sophisticated listening skills that identified implicit as well as explicit cues regarding client priorities, goals, and problems. (14)<br>…**fatigue diaries** and the development of comprehensive fatigue assessment tools were described by one participant as being useful strategies to make the invisible nature of fatigue more visible to the client, the family, and the healthcare team. (14)<br>For Anne, the formal support with exercise provided by the MS Therapy Centre was a way to adapt to the variability of MS, age and the need to be fit: I didn't find out about the MS Therapy Centre for a long, long time. Yeah… so. I suppose it came about at a time when I could fit it in with my life as well. (16)<br>…participants indicated that they found it useful to attend sessions with a neurological physiotherapist, who would have an appropriate environment for exercising and also adapt exercises to suit their capabilities. (18) | |
| | Social support<br><br>Red: who supported them | Definition of theme: This sub-theme identifies the value of family encouragement and social support in order to remain active.<br><br>Across the studies participants reported many examples of people who played a significant role in maintaining physical engagement (1,7,10,11,13,14,16,19). Family members (7,11,13,16,19), friends (11,13,16) exercise partners (1,13), health care providers(10,13,14), and regional MS societies (13,14,16) helped to overlap their physical, psychological or environmental barriers in relation to physical activity (1,7,10,11,13,14,16,19). For instance, Ben explained how his spouse signed him up for an exercise programme 6 years ago and since then he has remained active (7). In addition, Ruth stated: "… it has to be done within the family that you need support if you want to succeed at it" (19).<br><br>**VERBATIM STATEMENTS**<br>For some participants, the social connections they made participating in exercise, such as team sports, group exercise classes, and swimming squads, were motivating factors for exercise: "I've made great friends doing Pilates." (P1.2, 65 y, mod).(1)<br>At the same time, the spouses were cognizant of the important role they themselves could play in terms of facilitating their spouses' involvement in exercise and helping to overcome isolation. (7)<br>Ben commented that it was his spouse (Louise) who took the initiative by signing him up for an exercise program, and he has now been exercising for more than 6 years. "If she hadn't have signed us up for the program, I likely would not have gotten involved. (7)<br>Participant #13 was motivated by one particular in-home nursing aid who provided support, such as moving a portable mini-cycle by her chair and helping her perform stretches. (10)<br>…these women were able to self-manage their condition in relation to physical activity was through social support. In speaking with this group, it became quite apparent how integral their friends and family were in providing necessary support. (11)<br>One participant described how support of an exercise partner with MS helped her to stay motivated, and how exercise sessions were reduced for both if either experienced an exacerbation of symptom. (13) | 1,7, 10, 11, 13, 14, 16, 19 |

| | | | |
|---|---|---|---|
| | | They noted, however, that with help from friends, family and health care providers, they had been able to 'regroup' and resume their exercise programme. (13)<br>…. some regional MS societies, gym memberships, exercise classes, and transport are funded, and while these barriers are removed… (14)<br>HCP participants recognized the value of social support for people with MS-related fatigue seeking interventions such as exercise. (14)<br>Support provided by family, friends and the MS Therapy Centre in engaging in exercise and physical activity were viewed as a way of coping with MS. (16)<br>So it was very much, bringing us back together, we'll say, and showing that it has to be done within the family that you need support if you want to succeed at it' (Ruth). (19) | |
| | Self-management/ Control over fatigue<br><br>Colour scheme:<br>Red: self-management<br>Blue: effects<br>Pupil: inactivity effects<br>Yellow: techniques of self-management<br>Dark red: threads | Definition of theme: This sub-theme identifies the outcomes of self-management on perceived effects of physical activity on fatigue. It includes personalised strategies and their benefits.<br><br>Participants' exercise experience might resulted in a higher sense of responsibility for managing their symptoms (10,19) and empowered them to make decisions in regards to engaging in physical activity for increasing their control over fatigue (10,14,19). MS individuals adopted individualised problem-solving techniques (10,12,13,15) which enabled them to remain active (10). Their strategies were based on experimentation by listening to "their bodies" (12,14) and depended on their beliefs. Some were confident to exercise beyond the "edge" controlling the perceived fatigue with rest-periods and rehydration (12). Others chose safer strategies and made small gradual steps to avoid overdoing and turning back (13). Male participants appeared to "intuitively use self-regulation as a survival mechanism" (15). They chose the most suitable strategy for them without following specific guidance from professionals and sometimes exercised independently in order to control tiredness (15). One of them observed that: " complete rest after these exhausting periods was not helpful, and if he could motivate himself to go for a swim or do some exercise, he could get through that feeling of fatigue" (15).<br>Participants who achieved a high level of self-management, reported a strong sense of control over fatigue (10,12,14,15,19), were able to push themselves to their limits (12), adapted their energy requirements every moment (12), had self-efficacy, body awareness (14) and better perceived exercise outcomes (12).<br><br>**<u>VERBATIM STATEMENTS</u>**<br>The self-monitoring of PA behaviour and symptoms as well as the observed effects of PA on symptoms were important differences between groups. Active participants consistently wrote in their activity log and often noted problem-solving techniques that helped them engage in PA. (10)<br>Active participants often indicated that they were confident in managing their symptoms so that they could exercise, whereas inactive participants often indicated low self-efficacy. (10)<br>In this study, the level of control experienced by an individual seemed to play a pivotal role in perceived exercise outcomes. (12)<br>Participants who experienced a state of power sharing with fatigue described a strong sense of perceived control over fatigue and were able to adapt energy requirements on a moment by moment basis. These participants had moved | 10, 12, 13, 14, 15, 19 |

| | | | |
|---|---|---|---|
| | | beyond experimentation with various strategies and knew, by listening to their bodies, when it was appropriate to utilise a certain strategy. (12)<br>'The edge' was determined by both duration and intensity of exercise and also by utilisation of fatigue control strategies including rest periods and rehydration. (12)<br>I was able to control it more I was able to push myself to what my limit was; (12<br>…you . . . understand this on a deep level not just superficially and 'it's about setting a baseline for what you can do be it your mobility, and then what you do is take very gradual steps . . . and you really watch what 'you're doing in a day so that 'you're not overdoing it because if you overdo it you will go completely backwards . . . She said the way to do it is to do very small steps and very gradually over time you would increase the amount of activity your doing like walking . . . and that was a turning point for me because I understood. (13)<br>Client empowerment was regarded by several participants as a key part of their role and included, specifically, increasing levels of control, self-efficacy, choice, body awareness, and self-monitoring in clients:. . . being encouraged to respect the fact that it's their bodies and they know what they're feeling is truly what they are feeling, and through that having some empowerment that they can make decisions, the right decisions for that point in time. (Participant 7) (14)<br><br>…most of the men eventually took a flexible, problem-solving approach to managing their symptoms in the on text of exercise and were able to regain a sense of control. This approach, which appeared to be independent of any support from health care professionals… (15) One participant worked at home and found this useful because he could exercise and work "at his own pace." This helped him control tiredness. (15) He found that complete rest after these exhausting periods was not helpful, and if he could motivate himself to go for a swim or do some exercise, he could get through that feeling of fatigue. (15) . The men in this study appeared to intuitively use self-regulation as a survival mechanism. (15)<br><br>Joan goes on to describe how this experience enabled participants to feel, 'in control of it.' (19)<br>Participants described feeling a greater sense of personal responsibility for managing their fatigue. (19) | |
| | Patients' education<br><br>Colour scheme:<br>Red: learning information<br>Blue: outcome<br>Yellow: well known<br>Green: lack of willing | Definition of theme: This sub-theme identifies the perceived benefits from MS individuals' education about the fatigue management, exercise effects and an suitable introduction to physical activity.<br><br>Across the studies participants reported their learning about fatigue management (8,19) and exercise effects (5,8) as beneficial and some of them described that this new information was useful and might be applied elsewhere (9). Researchers attempted to give a deeper knowledge to their participants than might have achieved with MS society simple message "use it or lose it" (8) or the short advice for exercise repetition have been given by health care providers or gym instructors (13). They were focused in altering previous thinking about fatigue and guilty emotions around it, through the social & medical legitimisation of fatigue (19). In addition, other participants explored the mystery about exercise and felt stronger, gained confidence and knowledge and stated that: "guided exercise in the initial stages was the key" (5). However, some participants even though they had adequate knowledge about exercise benefits, "avoided pushing themselves beyond their limits" (11).<br><br>**<u>VERBATIM STATEMENTS</u>** | 5, 8, 9, 11, 13, 19 |

| | | | |
|---|---|---|---|
| | | Another participant said, "For me, I think it was taking the mystery out of exercise and giving the confidence that it's safe to do this and you'll get benefit from it and I think guided exercise in the initial stages was the key" (5)<br>… this is a clear message given by the local MS Society, as this person articulates: 'There's a very good phrase that is often used by other people, they use it as well [the MS society], use it or lose it. And that is really true with MS. If you don't use it you will lose it. There's no might or could be, there is you will'. (8)<br>Participants acknowledged that they had learned new things, both about their symptom-management and the normal effects of exercise…. Some participants described how they used what they had learned in the class and applied it elsewhere, . . . in the kitchen when you're at the work top, again you can add a wee exercise. (9)<br>It was clear when speaking to these women that the benefits of physical activity were well known by them and served as a motivating factor for being physically active, but they avoided pushing themselves beyond their limits. (11)<br>Use of set distances or times was based on advice from health care providers and gym instructors;… (13)<br>Legitimisation and validation was achieved through: participating in a programme that focused exclusively on fatigue management, through medical legitimisation of fatigue and through social legitimisation of fatigue as significant others were educated about fatigue. The experience of legitimisation was described as altering participants' previous experiences of guilt about their fatigue. (19)<br>All of the participants described altered thinking about their fatigue as a consequence of learning newly acquired knowledge. Education on a wide variety of options was provided: 'It covered everything from the medical point of view and we [also] had alternative therapies'. (19) | |
| | Rest & Pleasant activities<br><br>Colour scheme:<br>Red: planning rest<br>Yellow: Rest when necessary<br>Green: pleasant activities<br>Blue: effects | Definition of theme: This sub-theme identifies the perceived benefits of planning rests or using them when necessary, as a coping strategy to manage fatigue effects. Participants also chose to have some breaks with pleasant activities to restore their physical energy.<br><br>Across the studies participants were planning their activities such that they were having rests between them (1,11,12,15,18). Health care professionals often suggested them to break up activities (12) under planning (1,11,12,17,18,19), as a cessation during exercise (17) or "in response to the situation" (17,18). Participants reported as beneficial the use of short breaks with or without a cup of tea, in order to recover within an activity for reduction of fatigue symptoms (1,12,15). Female participants explained how they spread out their household activities over several days (11,18) to have a rest and avoid pushing themselves to their limits (11). Another strategy used for restoring physical energy was the undertaken of diversional or pleasant activities to distract their attention from the distress of experienced fatigue (17). For instance, a participant described: "Stretching out on the couch and listening to classical music ... I just love it. It is like eating chocolate with whipped cream." (17).<br><br>**VERBATIM STATEMENTS**<br>Participants prioritized and scheduled exercise, exercised in small increments, focused on exercise they enjoyed doing, employed self-monitoring techniques using apps and devices, made a commitment to exercise (such as buying a dog that had to be walked), and where necessary, made modifications to their regimens in order to maintain some level of exercise. (1) | 1,11,12, 15, 17, 18 |

| | | | |
|---|---|---|---|
| | | Throughout these interviews, many of the women described the adjustments they had made to their lifestyles since their diagnosis, especially when it came to doing any activity in relation to housework. Several of the women described having to spread out their homemaking tasks over a number of days, to allow for adequate rest in between tasks and to avoid pushing themselves too far beyond their limits. (11)<br>In addition, pacing strategies which alternated activity and rest were used in this state, often following advice from health-care professionals…. I'll start doing something, go for about half an hour, then sit down and have a cup of tea then get up and do something else. (12)<br>He took small breaks when he might "sit down and have a cup of tea" to recover before he continued with an activity. One man described how he could manage his physical activity if he did things slowly, as doing things quickly "brings it [the fatigue] on really quickly." Exercising with frequent breaks and at a slower pace or intensity seemed to reduce complex fatigue symptoms for men with MS. (15)<br>Strategies used to restore physical energy and/or attentional ability are recharge efforts. These included diversional activities, rest, and medications. Diversional activities were often used to distract attention from the distress of the fatigue experience and included listening to music, light reading, and talking on the telephone with friends. One participant said, "Stretching out on the couch and listening to classical music . . . I just love it. It is like eating chocolate with whipped cream." Another woman noted, "I visualize the glee and laughter of children... If I can pull a pleasant memory, it is like a mini-vacation." Rest was a planned or necessary cessation of physical activity… (17)<br>Common examples included planning not to undertake too many activities in one day and planning their schedules to allow rest in between activities. Participants commonly reported planning to complete activities in the morning and to rest in the afternoon. (18)<br>Break up activities over the course of the day or over several days, reference was made particularly to household activities Sometimes breaking up activities was planned. At other times, it was used in response to the situation. (18) | |
| | Heat & humidity/ Cooling strategies<br><br>Red: stay cool<br>Yellow: benefits | Definition of theme: This sub-theme identifies the implementation of different cooling methods in order to remain active when the weather is hot.<br><br>Across these studies MS individuals depicted their need to implement a variety of cooling strategies (10,13,15,17,18) to reduce the negative influence of hot weather on perceived fatigue, during exercise (15). The use of simple cooling methods as for example putting a wet towel round the neck (10), the use of ice cubes or cold water (17,18), reduce body temperature and increase their capacity for exercising (13,15,18). For instance, one woman stated: "I need my head to be cool ... . if it's real hot weather, I'll come in and put water on my head" (17). Other methods were mentioned, including exercise in air-conditioned environment or choosing to exercise the less warm hours of the day for preventing overheating (18).<br><br>**VERBATIM STATEMENTS**<br>Participant #1 wrote: 'I have kept a wet towel on my neck to stay cool. (10)<br>…cooling strategies during exercise were also important for women, who described the negative impact of hot and cold extremes on their capacity for exercise. (13) | 10,13,15,17, 18 |

| | | | |
|---|---|---|---|
| | | Although heat sensitivity is a well-recognized symptom of MS and several authors have already demonstrated the benefits of cooling strategies (Schwid et al., 2003), and this man offered additional insight into the potential for cooling strategies to increase perceived control over fatigue and exercise: (15)<br>Staying cool and efforts to get cool after over-heating are methods of controlling temperature. One man during the hot Texas summer said, "I have hidden [from the heat]. I have hibernated. We keep it cool in the house.., and if I don't keep it pretty frigid at night, I just don't sleep very well." A woman related, "I need my head to be cool . . . . if it's real hot weather, I'll come in and put water on my head." (17)<br>… participants reported using ice blocks, running hands and wrists under cold water, accessing air conditioning, and using neck wraps with ice inside. Also, they discussed choosing to undertake activities at certain times of the day, such as morning or late afternoon, when the air was cooler. (18) | |
| | Enjoyment from exercise<br><br>Red: Enjoyment to exercise<br>Blue: effects<br>Pupil: Having to exercise | Definition of theme: This sub-theme identifies the need of MS individuals to enjoy their physical activities in order to remain active.<br><br>Across these studies MS individuals and health care professionals highlighted the significance of enjoyment to participating in exercise activities as facilitator to remain motivated (1,5,9,14). For instance, a participant stated: "I've tried to keep the willpower in my head and it's working because I do enjoy it" (5). However, every patient perceived the benefits of diverse types of exercise differently and wanted to choose what suited best to his temporary individual needs (1). In addition, the imperative character of exercising led MS individuals to explore further a variety of physical activities in order to find an enjoyable type of exercise which kept a balance between the perceived drawbacks and benefits of exercise (1).<br><br>**<u>VERBATIM STATEMENTS</u>**<br>Exercising for pleasure contrasted sharply with "having" to exercise. Some had not found a type of exercise that was enjoyable despite trying different things, or their preferences changed over time: "I don't like gyms." (P3.2, 57 y, mild); "I don't like sports in general." (P5.2, 44, mild), "I suppose I've tried different forms of exercise… At the time I was doing swimming; I've done yoga as well. Yoga I really enjoyed but I'm not doing much of it at the moment… So I have experimented a bit with exercise to find the right thing." (P6.3, 39 y, mild). (1)<br>Participants prioritized and scheduled exercise, exercised in small increments, focused on exercise they enjoyed doing, employed self-monitoring techniques using apps and devices, made a commitment to exercise (such as buying a dog that had to be walked), and where necessary, made modifications to their regimens in order to maintain some level of exercise. (1)<br>For some, enjoyment of participating in exercise enhanced their motivation: "I've tried to keep the willpower in my head and it's working because I do enjoy it" (5)<br>I've found it really enjoyable, and I've been shocked that an improvement could be made by exercising at a much gentler level. (B10) (9)<br>…clients needed to enjoy exercise activities in order to remain motivated. (14) | 1,5, 9, 14 |
| | Weight management | Definition of theme: This sub-theme identifies how weight management works as motivator for some individuals to remain active. | 10, 11, 13 |

| | | | |
|---|---|---|---|
| | No Colour scheme | Across these studies MS individuals reported their fear of gaining weight if their physical activities will be further reduced (13) so, they grasped the chance to exercise hopping to lose weight (10,11).<br><br>**<u>VERBATIM STATEMENTS</u>**<br>The other most common incentive stated by participants was the desire to meet their personal health goals, such as losing weight, maintaining flexibility, and preventing cardiovascular disease. (10)<br>….two other women reported that they engaged in physical activity to lose weight. (11)<br>Fear of weight gain featured in participant descriptions: one participant strongly associated weight gain with reliance on a wheelchair, and another with growing dependency on others. (13) | |

(1) Barnard 2018, (2) Borkoles 2007, (3) Brown 2012, (4) Clarke 2015, (5) Crank 2017, (6) Dodd 2006, (7) Horton 2015, (8) Kayes 2011, (9) Learmonth 2012, (10) Plow 2009, (11) Schneider 2018, (12) Smith 2009, (13) Smith 2011, (14) Smith 2013 ,(15) Smith 2015 , (16) Stennett 2018, (17) Stuifbergen 1997, (18) Turpin 2015, (19) Twomey 2010.
